# Regulation of Nitrogen Fixation in *Bradyrhizobium* sp. Strain DOA9 Involves Two Distinct NifA Regulatory Proteins That Are Functionally Redundant During Symbiosis but Not During Free-Living Growth

**DOI:** 10.3389/fmicb.2018.01644

**Published:** 2018-07-24

**Authors:** Jenjira Wongdee, Nantakorn Boonkerd, Neung Teaumroong, Panlada Tittabutr, Eric Giraud

**Affiliations:** ^1^School of Biotechnology, Institute of Agricultural Technology, Suranaree University of Technology, Nakhon Ratchasima, Thailand; ^2^Laboratoire des Symbioses Tropicales et Méditerranéennes, Institut de Recherche Pour le Développement (IRD), UMR IRD, SupAgro, INRA, CIRAD, Université de Montpellier, Montpellier, France

**Keywords:** NifA, *Bradyrhizobium*, symbiosis, legume, nitrogen, nitrogenase, Rhizobium

## Abstract

The *Bradyrhizobium* sp. DOA9 strain displays the unusual properties to have a symbiotic plasmid and to fix nitrogen during both free-living and symbiotic growth. Sequence genome analysis shows that this strain contains the structural genes of dinitrogenase (*nifDK*) and the *nifA* regulatory gene on both the plasmid and chromosome. It was previously shown that both *nifDK* clusters are differentially expressed depending on growth conditions, suggesting different mechanisms of regulation. In this study, we examined the functional regulatory role of the two *nifA* genes found on the plasmid (*nifAp*) and chromosome (*nifAc*) that encode proteins with a moderate level of identity (55%) and different structural architectures. Using *gusA* (β-glucuronidase) reporter strains, we showed that both *nifA* genes were expressed during both the free-living and symbiotic growth stages. During symbiosis with *Aeschynomene americana*, mutants in only one *nifA* gene were not altered in their symbiotic properties, while a double *nifA* mutant was drastically impaired in nitrogen fixation, indicating that the two NifA proteins are functionally redundant during this culture condition. In contrast, under *in vitro* conditions, the *nifAc* mutant was unable to fix nitrogen, and no effect of the *nifAp* mutation was detected, indicating that NifAc is essential to activate *nif* genes during free-living growth. In accordance, the nitrogenase fixation deficiency of this mutant could be restored by the introduction of *nifAc* but not by *nifAp* or by two chimeric *nifA* genes encoding hybrid proteins with the N-terminus part of NifAc and the C-terminus of NifAp. Furthermore, transcriptional analysis by RT-qPCR of the WT and two *nifA* mutant backgrounds showed that NifAc and NifAp activated the expression of both chromosome and plasmid structural *nifDK* genes during symbiosis, while only NifAc activated the expression of *nifDKc* during free-living conditions. In summary, this study provides a better overview of the complex mechanisms of regulation of the nitrogenase genes in the DOA9 strain that involve two distinct NifA proteins, which are exchangeable during symbiosis for the activation of *nif* genes but not during free-living growth where NifAc is essential for the activation of *nifDKc*.

## Introduction

Rhizobium-legume symbiosis is considered as the major contributor of biologically fixed nitrogen to terrestrial ecosystems. The reduction of atmospheric N_2_ is catalyzed by the nitrogenase enzyme complex, which requires high-energy input in the form of ATP and electrons to break the triple bond. In addition, this enzymatic complex is highly sensitive to molecular oxygen, which irreversibly inactivates the enzyme. Diazotrophic bacteria have evolved sophisticated regulatory circuits of their nitrogen fixation (*nif*) genes in response to oxygen and nitrogen availability to prevent unnecessary energy consumption and permit the synthesis of the nitrogenase complex only during the proper environmental conditions (Burris and Roberts, 1993; Fischer, 1994).

A master regulator of nitrogen fixation is the NifA protein, which acts in association with the RNA polymerase sigma factor RpoN (σ^54^) to activate the expression of *nif* genes by binding to an upstream activating sequence (UAS; 5′-TGT-N_10_-ACA-3′). The NifA proteins show a typical three-domain structure. The N-terminal GAF domain is a ubiquitous signaling motif found in signaling and sensory proteins from all three kingdoms of life (Ho et al., 2000). The central domain interacts with the σ^54^-RNA polymerase and possesses ATPase activity, while the C-terminal domain shows a helix-turn-helix (HTH) motif involved in DNA-binding. The activity of NifA is directly sensitive to molecular oxygen, and in some cases, is also directly affected by combined nitrogen (Kullik et al., 1989; Souza et al., 1999; Steenhoudt and Vanderleyden, 2000). In addition, the *nifA* gene is subjected to transcriptional regulation, although the mechanisms vary depending on the rhizobial strain. For example, in *Sinorhizobium meliloti, nifA* expression is activated by the FixLJ two-component regulatory system in response to low oxygen tension, while in *Bradyrhizobium japonicum*, the *fixR-nifA* operon is controlled by the redox-responsive two-component system RegSR (Bauer et al., 1998).

The rhizobia generally display only one *nifA* copy, but one exception has been described for *Mesorhizobium loti*, which contains two *nifA* genes, *nifA1* and *nifA2*, both located on the symbiotic island (Nukui et al., 2006). The *nifA1* gene is most similar to the *nifA* of *Rhizobium etli, R. leguminosarum*, and *S. meliloti*, and it is found in an identical genomic context associated with other *nif* genes, while *nifA2* is most similar to *nifA* from *B. japonicum* and is not found in the vicinity of known *nif* genes (Sullivan et al., 2013). Interestingly, the two *nifA* genes are not functionally redundant, since the *M. loti nifA2* mutant form nodules that do not fix nitrogen, while the *nifA1* mutant is not affected symbiotically (Nukui et al., 2006).

Another example of the presence of two *nifA* genes has recently emerged with the analysis of the genome sequence of the non-photosynthetic *Bradyrhizobium* sp. DOA9 strain (Okazaki et al., 2015). This bacterium, isolated from rice paddy fields using *Aeschynomene americana* as a trap legume (Noisangiam et al., 2012), displays several unusual properties. First, unlike all described bradyrhizobia, this strain contains a symbiotic megaplasmid (pDOA9) that harbors *nod* and *nif* genes (Okazaki et al., 2015). Second, on both the chromosome and the plasmid, it can be distinguished the nitrogenase *nifHDK* genes that encode the subunits of the nitrogenase complex. In both cases, the *nifHDK* genes are split into two clusters, *nifH* and *nifDK* (Okazaki et al., 2015). Third, as described for photosynthetic bradyrhizobia, the bacteria that do contain a chromosomal *nifV* gene can fix nitrogen in both the free living and symbiotic states (Wongdee et al., 2016). Data from previous research indicated that both *nifDK* clusters contribute to nitrogenase activity during symbiosis with *A. americana*, while the *nifDK* cluster found on the chromosome is the major contributor to the nitrogenase activity of the bacteria under free-living conditions (Wongdee et al., 2016). These data indicate that the two *nifDK* clusters identified in the DOA9 strain should be differentially regulated. This is supported by the fact that the DOA9 display two *nifA* genes but also two *rpoN* homologous genes, both located on each replicon. The simple explanation is that the *nifA* found on the chromosome (*nifAc*) and the *nifA* found on the plasmid (*nifAp*) specifically regulated the *nifDK* cluster found on the replicon where *nifA* is present. However, crosstalk between these two regulatory circuits would also be expected, given that NifDKp proteins require the expression of the accessory *nif* genes to form a functional nitrogenase. In particular, the *nifENX* genes whose products are needed for synthesis of the iron-molybdenum cofactor of nitrogenase, exist as a unique copy and are found downstream of the *nifDKc* cluster.

Thus, in the present work, we aimed to investigate in more detail the regulatory functions of the two *nifA* genes identified for the *Bradyrhizobium* sp. DOA9. In a first approach, we analyzed the expression levels of both *nifA* genes under different culture conditions using translational fusions to *gusA* (β-glucuronidase). We then analyzed the contribution of each regulatory protein to the control of bacterial nitrogenase activity under free-living and symbiotic states by constructing single and double *nifA* mutants. Finally, the expression level of several *nif* genes in three different backgrounds, the DOA9 wild-type (WT), Δ*nifAc*, and Δ*nifAp* mutant strains, were analyzed to identify which genes are activated by NifAc and NifAp.

## Materials and Methods

### Bacterial Strains and Culture Media

The *Bradyrhizobium* sp. DOA9 WT was obtained from the School of Biotechnology, Suranaree University of Technology, Thailand, while all mutants were constructed in the Laboratoire des Symbioses Tropicales et Méditerranéennes (LSTM), France. These bacterial strains were grown at 28°C for 4 days in Yeast extract-mannitol (YM) medium (Vincent, 1970) or a BNM-B minimal medium (Renier et al., 2011). *Escherichia coli* strains were grown in LB medium at 37°C. When required, the media were supplemented with the appropriate antibiotics at the following concentrations: 100 μg/ml kanamycin, 200 μg/ml streptomycin, 20 μg/ml nalidixic acid, and 20 μg/ml cefotaxime.

### Construction of the Reporter and Mutant Strains

All DNA fragments were amplified using the primers listed in **Supplementary Table S1**. To construct the reporter strains, DOA9-*Pm-fixRnifAc* and DOA9-*Pm*-*nifAp*, the 500-bp upstream region of *fixR* and the *nifAp* operon were amplified by PCR and cloned into the plasmid pVO155-*npt2-cefo-npt2-gfp*. This plasmid, which is a derivative of the pVO155 plasmid (Oke and Long, 1999), could not replicate in the *Bradyrhizobium* strains. The plasmid carries the promoterless *gusA, gfp*, kanamycin, and cefotaxime genes under the constitutive promoter *npt2* (Okazaki et al., 2016). To construct the two DOA9*ΩnifA* (insertion) mutants, 300 to 400 base pairs (bp) of the internal sequence of each *nifA* gene were amplified by PCR and cloned into the plasmid pVO155-*npt2-cefo-npt2-gfp*. To construct the DOA9Δ*nifA* deletion mutants, the upstream and downstream regions (between 700 and 1000-bp) of each *nifA* gene were amplified and merged using overlap extension PCR. Then, the fragment was cloned into the plasmid pK18 mob-*cefo-sacB*. This plasmid carries the *sacB* gene, which induces bacterial death in the presence of sucrose and the kanamycin-resistance gene (Tsai and Alley, 2000) as well as the cefotaxime gene under the *npt2* promoter that was added to the *KpnI* site. The various constructed plasmids were transferred into the DOA9 strain by mating, followed by the insertion or deletion of the selected mutants as previously described (Wongdee et al., 2016).

### Complementation of the DOA9Δ*nifAc* Mutant

For the DOA9Δ*nifAc* mutant, the complete *nifAc, nifAp*, or hybrid of *nifAc* and *nifAp* genes were amplified and cloned downstream of the *npt2* promoter into the pMG103-*npt2-cefo* plasmid that harbored a cefotaxime resistance gene (Wongdee et al., 2016). This plasmid is stable and replicative in the DOA9 strain. To construct the hybrid *nifA* genes, the 5′-region of *nifAc* and the 3′-region of *nifAp* were amplified and merged using overlap extension PCR. The constructed plasmids were introduced into the competent cells of the DOA9Δ*nifAc* mutant by electroporation. The complemented strains were selected on YM plates supplemented with 20 μg/ml cefotaxime and 20 μg/ml nalidixic acid.

### Plant Cultivation and Analysis Under Symbiotic Condition

The symbiosis efficiency of the *Bradyrhizobium* DOA9 strain and its derivatives were tested with *A. americana* No. 281 collected from the LSTM greenhouse. The seeds were surface sterilized by immersion in sulfuric acid under shaking for 45 min. Seeds were thoroughly washed with sterile distilled water and incubated overnight in sterile water. Seeds were transferred for 1 day at 37°C in the darkness on 0.8% agar plates for germination. Plantlets were transferred onto the top of the test tubes and covered by aluminum paper for hydroponic culture in buffered nodulation medium (BNM) (Ehrhardt et al., 1992). Plants were grown in a 28°C growth chamber with a 16-h light and 8-h dark regime and 70% humidity. Seven days after transfer, each seedling was inoculated with 1 ml of cell suspension resulting from a 5-day-old bacterial culture washed in BNM and adjusted to an optical density of one at 600 nm. For nodulation and the nitrogen fixation assay, 10 to 20 plants per condition were taken at 20 days post-inoculation (dpi) and analyzed for the number of nodules and nitrogenase activity as previously described (Bonaldi et al., 2010). The experiments were performed in duplicate.

### Cytological Analysis

To follow the GUS activity in the nodules elicited by the reporter strains, 30- to 40-μm-thick sections from fresh nodule samples were prepared using a vibratome (VT1000S; Leica, Nanterre, France) and incubated at 37°C in the dark in GUS assay buffer for 1 h, as described in Bonaldi et al. (2010). After staining, the sections were mounted and observed under bright-field illumination with a macroscope (Nikon AZ100; Champigny-sur-Marne, France).

### Determination of Nitrogenase Activity Under Free-Living Conditions

To determine the nitrogenase enzyme activity under free-living conditions, the *Bradyrhizobium* sp. strain DOA9 and derivatives were grown in 10-ml test tubes hermetically closed (BD Vacutainer, Franklin Lakes, NJ, United States) containing 2 ml of semisolid BNM-B medium (agar 0.8% w/v). The BNM-B medium is a synthetic plant growth medium (Ehrhardt et al., 1992) supplemented with a carbon (10 mM succinate) and a cocktail of vitamins (riboflavin at 0.2 μg/ml, biotin at 0.12 μg/ml, thiamine-HCl at 0.8 μg/ml, myo-inositol at 0.5 μg/ml, p-aminobenzoic acid at 0.1 μg/ml, nicotinic acid at 0.5 μg/ml, calcium pantothenate at 0.8 μg/ml, and cyanocobalamin at 1 ng/ml) to support growth of *Bradyrhizobium* strains (Renier et al., 2011). It is to note that the BNM-B medium was not supplemented with a nitrogenous source but the bacteria growth is possible thanks to the dinitrogen and oxygen present in the air constituting the initial headspace of the test tube. Just after closing hermitically the tubes, acetylene gas (1 ml) was injected to a final concentration of 10%. The cultures were then incubated at 28°C without shaking, and the gas samples were analyzed at 7 dpi for ethylene production by gas chromatography, as previously described (Renier et al., 2011).

### Determination of β-Glucuronidase (GUS) Activity Under Free-Living Conditions

The two DOA9 reporter strains were grown for 4 days in YM medium, collected, and washed with BNM-B medium as described above. Bottles of 150-ml sealed with rubber stoppers and containing 55-ml of BNM-B medium and 95-ml of air were then inoculated with DOA9 reporter strains to an initial OD_600_ of 0.05. The cultures were then incubated at 28°C without shaking. After 7 days, the bacterial culture was removed from the bottle, and GUS activity was measured using the substrate *p*-nitrophenyl glucuronide (PNPG) as described by Jefferson (1987). β-glucuronidase units were calculated according to Miller (1972).

### RNA Purification, cDNA Synthesis, and qRT-PCR

The expression of genes involved in nitrogen fixation of strain DOA9 was determined from cells grown under free-living conditions and bacteroids obtained from nodules of *A. americana* under symbiotic conditions. For free-living conditions, the bacterial cells were grown in 150-ml bottles as described just above. For harvesting, cultures were added to a 1:10 volume of “stop solution” [10% Tris-HCl-buffered phenol (pH 8) in ethanol], and cells removed from the liquid medium by centrifugation for 10 min (10,000 rpm, 4°C). The cell pellets were frozen in liquid nitrogen and stored at -80°C. Analysis under symbiotic conditions and RNA isolation from bacteroids were processed from approximately 1 g of frozen nodules by homogenization with a tungsten carbide bead (3 mm; Qiagen, Hilden, Germany) in 2-ml microcentrifuge tubes. Total RNA was isolated from the free-living bacterial cells, and the nodules were disrupted with a hot (65°C) phenol-extraction procedure that was previously described (Babst et al., 1996). RNA was purified and treated with DNase using mini-prep kits (Qiagen, Valencia, CA, United States). Then, the cDNA was synthesized with iScript TM Reverse transcription Supermix for RT-qPCR (Bio-Rad, Hercules, CA, United States). Then, 10–50 ng of each cDNA sample was added to PowerUP^TM^ SYBRTM Green master mixed buffer (Applied Biosystems, United States, Canada), and the appropriate amount of specific primers (listed in **Supplementary Table S2**) were used in the qRT-PCR analyses using an annealing temperature at 55°C for all reactions. The expression of target genes was relatively compared with the expression of the housekeeping gene, *dnaK*, using QuantStudio Design & Analysis Software from Applied Biosystems.

## Results

### *Bradyrhizobium* sp. Strain DOA9 Displays Two Distinct *nifA* Genes Located on Both Chromosome and Mega-Plasmid (pDOA9)

**FIGURE 1:**
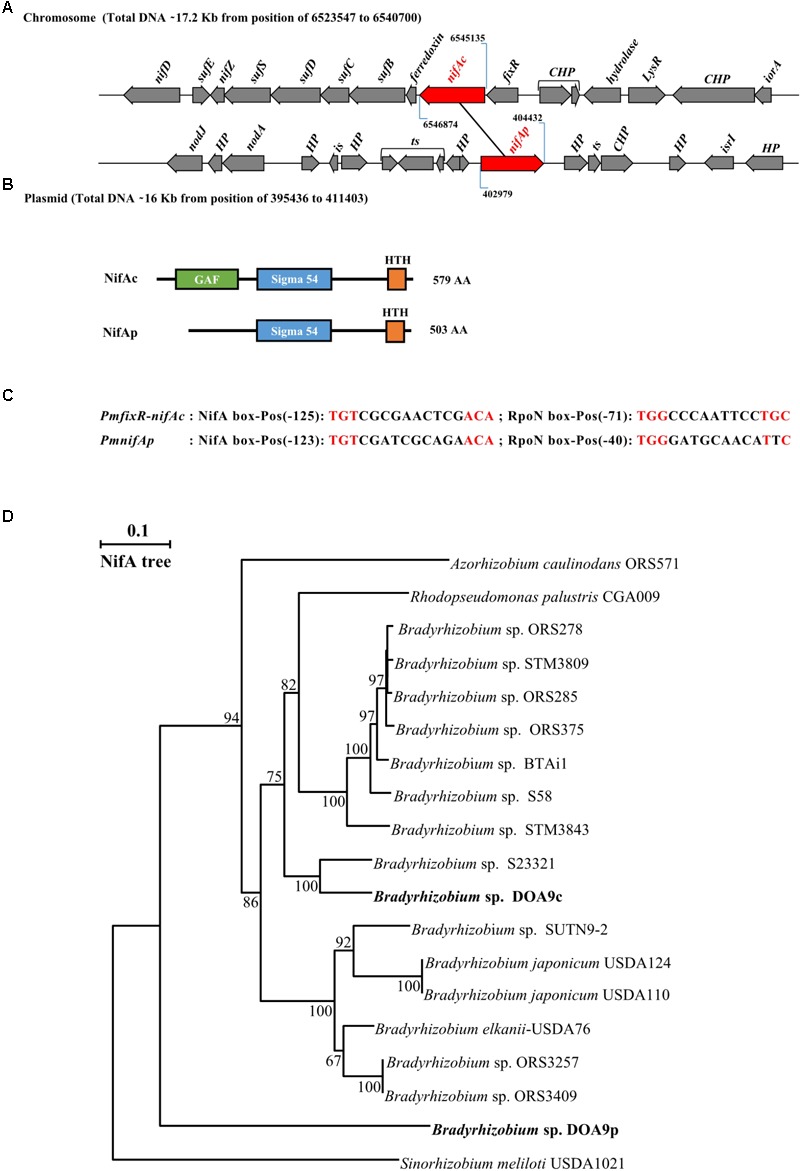
*Bradyrhizobium* sp. strain DOA9 strain displays two distinct *nifA* genes. **(A)** Genetic organization of the two *nifA* genes (in red) located on both the chromosome and plasmid of the DOA9 strain. *nifAc, nifA* located on the chromosome; *nifAp, nifA* located on the plasmid; CHP: Conserved Hypothetical protein, HP: Hypothetical protein, *is*: intregrase and *ts*: transposase. **(B)** Predicted domain structure of both NifA proteins. **(C)** Putative NifA and RpoN boxes identified in promoter region of *fixR-nifAc* operon and *nifAp*. Pos: position of 5′ end nucleotide of motif relative to annotated start codon. **(D)** NifA phylogenetic tree showing relationship between NifA of bradyrhizobia. Sequences were aligned by CLUSTALX, and the tree was generated using the neighbor-joining method (Saitou and Nei, 1987) and displayed using NJPLOT (Perrière and Gouy, 1996). Bootstrap values, expressed as percentages of 1000 replications, are shown at branching points.

Two *nifA* homologous genes can be identified in the *Bradyrhizobium* DOA9 strain. One copy found on the chromosome, termed *nifAc*, is located approximately 6 kb from the *nifDKENX* operon and found just downstream of the *fixR* gene (**Figure 1A**). In *B. japonicum, nifA* is also found downstream of *fixR*. It has been shown that the two genes are part of the same transcript (Thöny et al., 1987), suggesting that *fixR nifAc* also forms an operon in the DOA9 strain. Downstream of this operon, a gene (*fer*) encoding a 4Fe-4S ferredoxin and a *suf* operon composed of four genes (*sufB, sufC, sufD*, and *sufS*) were identified and have been shown to function in the assembly of iron-sulfur clusters (Takahashi and Tokumoto, 2002). The other copy found on the plasmid, known as *nifAp*, is surrounded by genes of unknown function, and no known *nif* or *fix* genes are found in the vicinity. The two corresponding NifA proteins are clearly distinct and are of different lengths; NifAc (579 aa) and NifAp (503 aa) display only 52% identity. A Pfam analysis to identify functional domains showed that NifAc displays a classical NifA architecture with a N-terminal GAFdomain, a central sigma 54 interaction domain and a C-terminal HTHdomain. NifAp shows a less classical structure with only the presence of the central and HTH domains (**Figure 1B**). The divergence of NifAp is not limited to the absence of the N-terminal GAF domain, since phylogenetic analysis showed that this protein formed an outgroup that was well separated from the NifA proteins identified in *Bradyrhizobium* strains (**Figure 1D**).

Interestingly, in both cases, a close examination of the promoter regions of the *fixRnifAc* operon and *nifAp* permitted the identification by manual analysis of a putative NifA and a RpoN binding sites, suggesting that both NifA proteins could autoregulate their own expression level and that of their homolog (**Figure 1C**).

### Two *nifA* Genes Identified in DOA9 Strain Are Both Expressed During Symbiosis and Free-Living Growth

**FIGURE 2:**
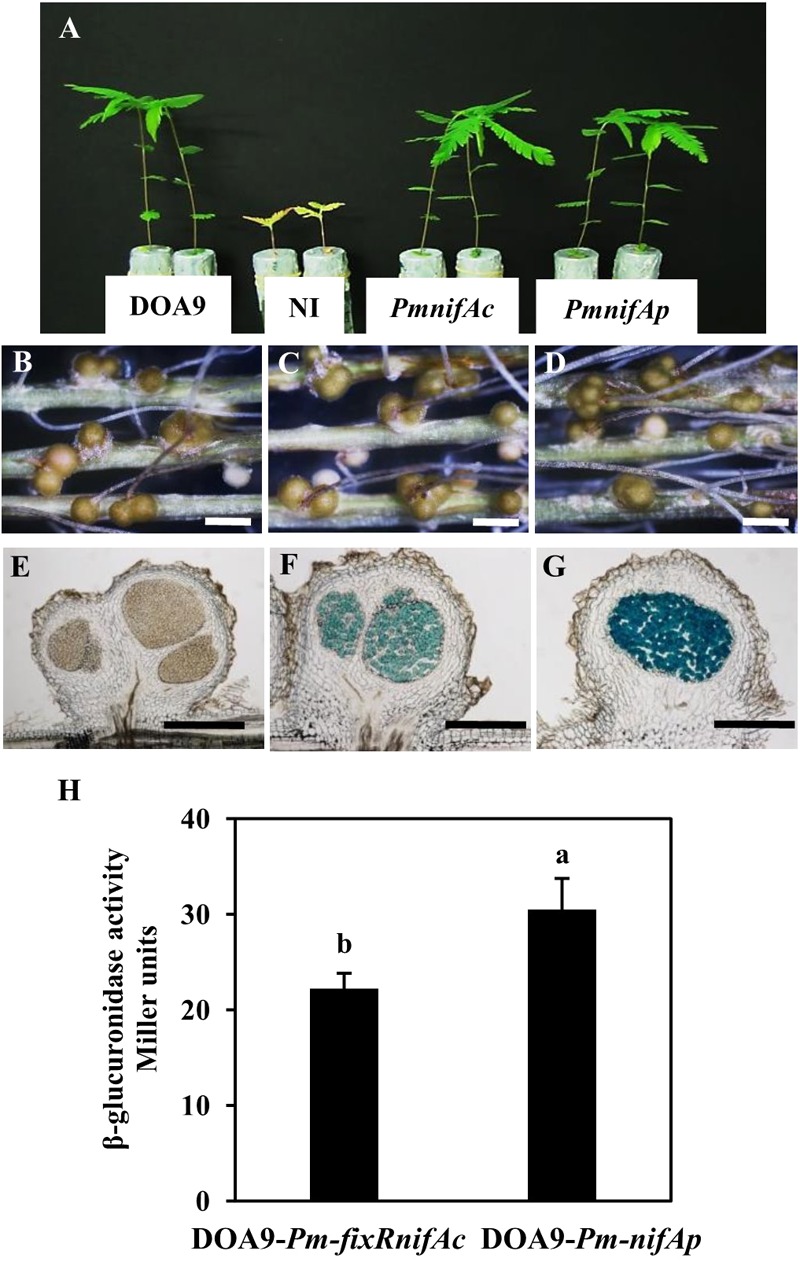
The two *nifA* genes of *Bradyrhizobium* sp. DOA9 strain are expressed during symbiosis with *Aeschynomene americana*. **(A)** Comparison of growth of plants (aerial part) non-inoculated (NI) or inoculated with WT and reporter strains DOA9-*Pm-fixRnifAc* and DOA9-*Pm*-*nifAp* (at 14 dpi). **(B–D)** Root nodules observed with a stereomicroscope stereomicroscope. **(B)** Nodules elicited by WT; **(C)** Nodules elicited by DOA9-*Pm-fixRnifAc*; **(D)** Nodules elicited by DOA9-*Pm*-*nifAp*. **(E–G)** Expression of *gusA* reporter gene revealed on 40 μm nodule sections stained with X-Gluc. Nodules elicited by WT **(E)**, DOA9-*Pm-fixRnifAc* **(F)** and DOA9-*Pm-nifAp* **(G)**. Scale bars are 1 mm for **(B–D)** and 250 μm **(E–G)**. **(H)**, β-glucuronidase activity of DOA9-*Pm-fixRnifAc* and DOA9-*Pm-nifAp* reporter strains grown after 7 days of culture under free-living conditions (microaerobiosis and not combined nitrogen source). Different letters above error bars indicate significant differences at *P* < 0.05 (Tukey’s HSD test).

To analyze the expression of the two *nifA* genes identified in the *Bradyrhizobium* DOA9 strain, we constructed two reported strains (DOA9-*Pm-fixRnifAc* and DOA9-*Pm-nifAp*) by integrating the nonreplicative plasmid pVO155-*npt2-cefo-npt2-gfp*, which carries a promoterless *gusA* gene (Okazaki et al., 2016) downstream of the promoter region of the *fixRnifAc* operon and *nifAp* gene. Since the DOA9 strain was isolated using *A. americana* as a trap, we analyzed these two reporter strains in this host plant. Observations at 14 dpi showed that both reporter strains were able to nodulate and fix nitrogen similar to the WT-strain, indicating that the integration of the pVO155 plasmid in these two promoter regions did not alter the symbiotic performance of the strain (**Figures 2A–G**). Cytological analysis revealed a β-glucuronidase activity in the nodules, which was elicited by the two reporter strains, in contrast to the WT-nodules for which no activity could be detected (**Figures 2E–G**). Although X-gluc (5-Bromo-4-chloro-3-indolyl-β-D-glucuronide cyclohexylamine salt) staining is a qualitative measurement of gene expression, the naked eye could observe that the nodules elicited by DOA9-*Pm-nifAp* displayed a more intense blue color than those elicited by the DOA9-*Pm-fixRnifAc* reporter strain (**Figures 2F,G**), which indicates slight differences in the expression of the two *nifA* genes.

Similar observations were also made during free-living growth under microaerobic conditions and the absence of a combined nitrogen source. Indeed, after 7 days of culture in these conditions, the β-glucuronidase activity measured for DOA9-*Pm-nifAp* (30 Miller unit) was higher than that detected for the DOA9-*Pm-fixRnifAc* reporter strain (22 Miller Unit) (**Figure 2H**). Taken together, these data indicate that the two *nifA* genes identified in the DOA9 strain are expressed during symbiotic and free-living conditions and that in both conditions, the level of expression of *nifAp* is slightly higher than that of *nifAc*.

### *nifAc* and *nifAp* Genes in *Bradyrhizobium* sp. DOA9 Strain Are Functionally Redundant During Symbiosis

**FIGURE 3:**
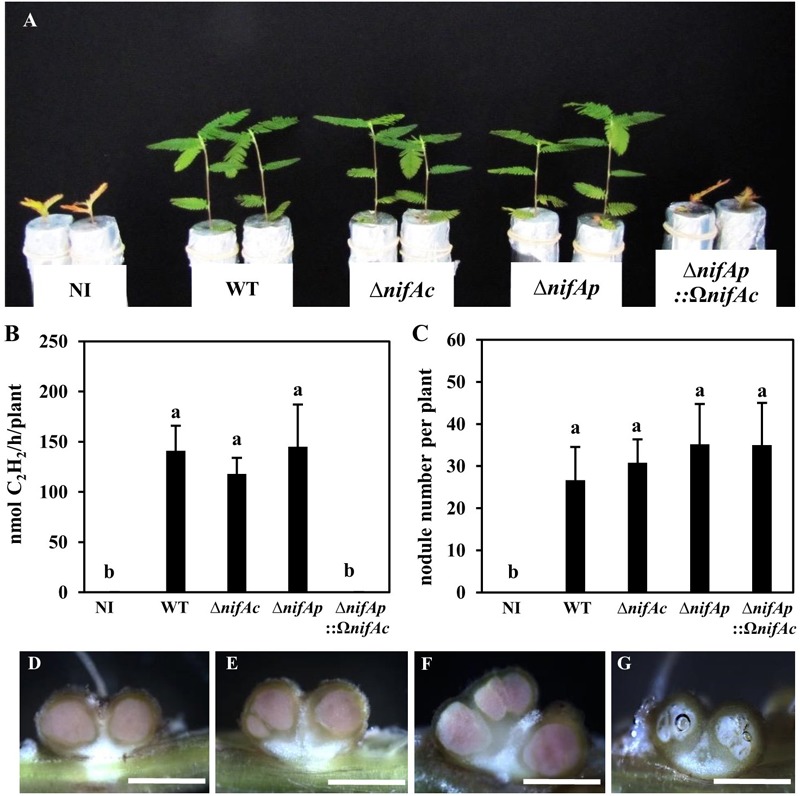
The two *nifA* genes in *Bradyrhizobium* sp. DOA9 strain are functionally redundant during symbiosis with *Aeschynomene americana.* **(A)** Comparison of plant growth (aerial part) non-inoculated (NI) or inoculated with WT and mutant strains DOA9*ΔnifAc*, DOA9*ΔnifAp* and DOA9*ΔnifAp*::Ω*nifAc*DOA9 (at 20 dpi). **(B)** Amount of acetylene-reducing activity (ARA) in *A. americana* plants inoculated with WT and mutant strains. **(C)** Number of nodules per plant inoculated by WT and *nifA* mutant strains **(D–F)** Transversal sections of nodules elicited by WT **(D)**, DOA9*ΔnifAc* **(E)**, DOA9*ΔnifAp* **(F)**, and DOA9*ΔnifAp*::Ω*nifAc* **(G)** mutants. Scale bars are 1 mm for **(D–G)**. In **(B,C)**, error bars represent standard error (*n* = 10). Different letters above error bars indicate significant differences at *P* < 0.05 (Tukey’s HSD test).

The NifA protein has been shown to be essential for symbiotic nitrogen fixation in several rhizobia (Szeto et al., 1984; Schetgens et al., 1985; Fischer et al., 1986; Iismaa and Watson, 1989). To appreciate the relative importance of each *nifA* gene identified in DOA9 during symbiosis, we constructed various *nifA* mutants, single *nifA* mutants, either by insertion (DOA9Ω*nifAc* and DOA9Ω*nifAp*) or deletion (DOA9Δ*nifAc* and DOA9Δ*nifAp*), and a double *nifA* mutant (DOA9Δ*nifAp*::Ω*nifAc*). As shown in **Figures 3A–C** and **Supplementary Figure S1**, the plants inoculated with the different single mutants displayed no significant difference from those inoculated with the WT strain in terms of their growth, the number of nodules formed or the measured nitrogenase activity indicating that the single mutation of the *nifAc* or *nifAp* gene had no impact on the symbiotic performance of the strain. In contrast, the plants inoculated with the double *nifA* mutant (*ΔnifAp*::Ω*nifAc*) displayed a strict fix minus phenotype (**Figure 3B**), and the growth of the plants was similar to that of the non-inoculated plants. Notably, the double *nifA* mutant induced nodules that were smaller and displayed symptoms of senescence (they were white instead of pink, indicating the absence of leghemoglobin and the central tissue was digested) (**Figures 3D–G**). Taken together these data suggest that the two NifA proteins identified in the DOA9 strain are functionally redundant during symbiosis and that at least one functional NifA protein is absolutely required for symbiotic nitrogenase activity, as observed in other rhizobia.

### NifAc Is Essential for Nitrogen Fixation Under Free-Living Conditions

To determine whether the two *nifA* genes were also exchangeable during free-living conditions, we analyzed the nitrogenase activity of the different constructed *nifA* mutants after 7 days of culture in semisolid BNM medium. The *nifAc* mutants including DOA9Ω*nifAc* and DOA9Δ*nifAc* were obviously unable to fix nitrogen in their free-living state (**Table 1**). In contrast, DOA9Ω*nifAp* and DOA9Δ*nifAp* mutants displayed nitrogenase activity similar to the WT strain. These data indicate that NifAc is essential for nitrogenase activity during the free-living condition, while NifAp does not play a significant role in this condition. As expected, it was found that the double *nifA* mutant was not able to fix nitrogen in the free-living state (**Table 1**).

**Table 1:** Nitrogenase activity in *Bradyrhizobium* sp. DOA9 (WT) and *nifA* mutant strains grown under free-living conditions as described in Material and Methods.

Treatments^∗^	Acetylene reduction	SD
	(nmol/h/culture)	
Non-inoculation	ND	ND
WT	3,643.55	±18.01
*ωnifAc*	ND	ND
*ΔnifAc*	ND	ND
*ωnifAp*	3,588.72	±74.99
*ΔnifAp*	3,547.54	±35.54
*ΔnifAp::ωnifAc*	ND	ND
*ΔnifAc:*:pMG103*:nifAc*	3,535.37	±137.4
*ΔnifAc:*:pMG103*:nifAp*	ND	ND
*ΔnifAc:*:pMG103*:nifAp hybrid1*	ND	ND
*ΔnifAc:*:pMG103*:nifAp hybrid2*	ND	ND

*^∗^Nitrogen-starved cells were inoculated into glass vials containing 2 mL of medium and 10 mL of gas headspace and incubated at 30°C for 7 days in semisolid culture. SD, standard deviation determined from three replicates; ND, non detected.*

Because NifAp lacks the N-terminal GAF domain, a simple hypothesis would be to postulate that NifAp protein is not active under free-living conditions, due to the absence of this functional domain. To check this hypothesis, we constructed two chimeric NifA hybrid proteins, one corresponding to the almost complete NifAp, to which has been added the first 70 AA of NifAc, the second corresponding to the sigma 54 interaction domain and HTH domain of NifAp (from the AA 150 to 503), to which was added the complete GAF domain of NifAc (the first 220 AA). These constructs were cloned into the pMG103 plasmid under the constitutive *npt2* promoter and reintroduced into the DOA9Δ*nifAc* mutant (**Supplementary Figure S2**). As controls, we also reintroduced the complete *nifAc* or *nifAp* gene using the same plasmid and *npt2* promoter. As shown in **Table 1**, only the reintroduction of the complete *nifAc* gene completely restored the nitrogenase activity of the DOA9Δ*nifAc* mutant. No gain of function was observed for all other constructs. This suggests that simple addition of the GAF domain is not sufficient to rebuild functional activity in NifAp protein under free-living conditions.

### NifAc and NifAp Activate Differently the Expression of Chromosome and Plasmid *nifHdk* Genes According to the Culture Conditions

The analysis of *nifA* mutants on plants suggested that the two NifA proteins are functionally redundant, but considering that a functional redundancy of the *nifHDK* genes found on the chromosome and the plasmid was also reported (Wongdee et al., 2016), we cannot completely exclude the possibility that the absence of phenotype observed for the single *nifA* mutants results in fact to this last redundancy. In other words, we can ask whether each NifA protein activates only one specific set of *nifHDK* genes, or both sets found on the chromosome and the plasmid.

**FIGURE 4:**
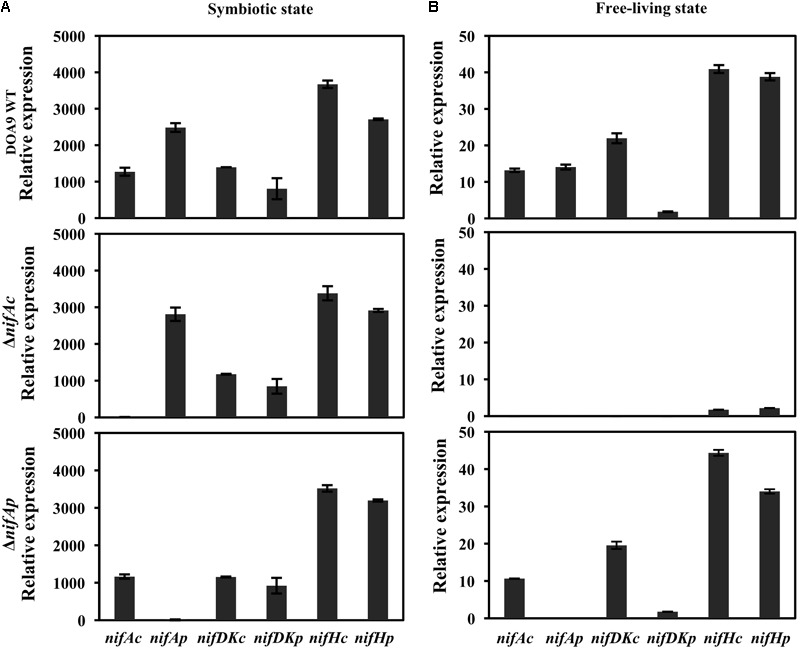
Expression of nitrogen-fixing genes (*nifDKc, nifDKp, nifHc*, and *nifHp*) and *nifAc, nifAp* genes in *Bradyrhizobium* sp. DOA9 (WT) and *nifA* mutant strains grown under symbiosis **(A)** or free-living conditions **(B)**. Bacteroid cells were obtained from *A. americana* nodules at 20 days post-inoculation **(A)**, and bacterial cells grown under free-living conditions were obtained after 7 days of culture in BNM-B medium without glutamate **(B)**. Total RNA was extracted and subjected to quantitative reverse transcription-PCR (qRT-PCR) with an internal standard of *dnaK*. All data were from one representative experiment that was repeated three times. Error bars indicate standard deviation.

To answer this question, we used quantitative reverse transcription PCR (qRT-PCR) to analyze the level of expression of the structural *nif* genes (*nifDKc, nifHc, nifDKp, nifHp*) as well as controls, namely, the two regulatory *nifA* genes (*nifAc, nifAp*) in both the WT and the two Δ*nifAc* and Δ*nifAp* mutants. As shown in **Figure 4A**, during symbiosis, the expression profiles of all structural *nif* genes is well conserved in the WT and the two *nifA* mutants. This clearly demonstrates that in this condition, the two NifA proteins can activate the expression of the two *nifHDK* sets, definitively confirming their functional redundancy in this condition. In contrast, during free-living growth, we observed that the Δ*nifAc* mutant differed drastically from the WT and the Δ*nifAp* mutant (**Figure 4B**). Indeed, in the Δ*nifAc* mutant, no expression of *nifDKc* and *p* was detected and the expression levels of *nifHc* and *p* were also extremely low. These data, which were in concordance with the absence of nitrogenase activity detected in this mutant, confirms the essential role of NifAc protein in the activation of *nif* genes under free-living conditions. In addition, for this mutant, no expression of *nifAp* could be detected, indicating that NifAc also activates the expression of *nifAp* which is in accordance with the presence of putative NifA and RpoN binding sites found in the *nifAp* promoter region.

In contrast, the pattern of expression of the *nif* genes remains very similar between the WT strain and the Δ*nifAp* mutant during free-living conditions, confirming that NifAp is dispensable during this condition. Interestingly, in these culture conditions, *nifDKc* was expressed at a far higher level than *nifDKp.* These data are in agreement with our previous study, which showed that NifDKc was the major contributor to the bacterial nitrogenase activity under free-living conditions (Wongdee et al., 2016).

Taken together, these data confirm that the two NifA proteins are exchangeable in the activation of *nif* genes during symbiosis, but not during free-living conditions, where NifAc is essential.

## Discussion

In this study, we showed that the two *nifA* genes present in the *Bradyrhizobium* sp. DOA9 strain are expressed during both symbiotic and free-living growth and encoded functional proteins. In particular, we observed that these two NifA are perfectly exchangeable for the regulation of nitrogen fixation during symbiosis. These data were unexpected, given the moderate level of identity between these two proteins and the difference observed in their architectural organization (see Discussion below). Furthermore, in the only other example rhizobial strain reported to contain two *nifA* genes, *Mesorhizobium loti*, there was no functional redundancy observed between these two NifA proteins since the Δ*nifA2* mutant gave a Fix^-^ phenotype, while no symbiotic defect was observed for the Δ*nifA1* mutant (Sullivan et al., 2001; Nukui et al., 2006). In fact, the regulatory role of NifA1 in this last bacterium is unclear, since the expression of NifA-regulated genes, i.e., those containing NifA and RpoN binding sites boxes in their promoter, were drastically impacted in the Δ*nifA2* mutant but not in the Δ*nifA1* background (Sullivan et al., 2013).

The origin of these two *nifA* genes in the DOA9 strain is puzzling. Their localization on different replicons and their moderate level of identity suggest that they were separately acquired, rather than via duplication of a single gene. In both cases, a Blast search using the amino acid sequence of NifAp or NifAc returned as best hits *Bradyrhizobium* NifA homologs, suggesting that both *nifA* genes derived from a common ancestor. However, while the percentage of identity of NifAc with the other bradyrhizobial NifA ranges between 91 to 76 %, this percentage drops to 55-50% for NifAp. In accordance, a phylogenetic analysis (**Figure 1D**) clearly showed that NifAp forms an outgroup from the bradyrhizobial NifA proteins. Furthermore, *nifAp* is found in an unusual genomic context, as no known *nif* or *fix* genes are found in the vicinity, in contrast to the other rhizobial *nifA* genes that were always found associated with genes involved in nitrogen fixation (Fischer, 1994). The presence of insertion sequence elements belonging to the IS3 family surrounding *nifAp* suggests the possibility that *nifAp* could have been separated from *nif* genes by a transposition event (**Supplementary Text S1**).

In all of the rhizobia in which *nifA* has been studied, it has been shown that NifA is absolutely required to activate nitrogen fixation during symbiosis (Szeto et al., 1984; Schetgens et al., 1985; Fischer et al., 1986; Iismaa and Watson, 1989). If the plant perceives that the nodules are ineffective, a sanctioning program is rapidly triggered (Westhoek et al., 2017), such as observed in this study for the double *nifA* mutant for which the nodules were senescing. Therefore, we can assume that NifA is essential for the symbiotic life of rhizobia and that there is a high selective pressure to maintain its functionality. DOA9 contains two *nifA* genes, and this likely relaxed this selective pressure and permitted a higher evolution rate of one homologous gene, i.e., *nifAp*. On the other hand, although *nifAp* has strongly diverged from the other *nifA*, NifAp remains functional, as it can activate both *nifHDKc* and *p* during symbiosis in the absence of NifAc. This suggests that maintaining two functional NifA proteins in DOA9 strain may be selectively advantageous even if there is an overlap in their regulatory function.

The major striking difference between NifAp and NifAc or the other bradyrhizobial NifA is the lack of an N-terminal GAF domain. There are several reports in various diazotrophic bacteria indicating that the GAF domain plays a key role in the modulation of NifA activity. For example, in *Azotobacter vinelandii*, the GAF domain binds 2-oxoglutarate, a key metabolic signal of carbon status, the presence of which influences the interaction with the antiactivator protein NifL (Martinez-Argudo et al., 2004). In the same vein, in *Herbaspirillum seropedicae*, NifA regulation by ammonium involves its N-terminal GAF domain and the signal transduction protein GlnK (Aquino et al., 2015). In contrast, to our knowledge, no functional role has been attributed to the N-terminal GAF domain of the rhizobial NifA proteins. It does not play an obvious role, since its deletion in the NifA of *S. meliloti* or *B. diazoefficiens* does not impair the ability of the protein to activate *nif* genes (Beynon et al., 1988; Fischer et al., 1988). Furthermore, it exists among rhizobia, one example (*R. leguminosarum)*, for which NifA naturally lacks this GAF domain and which, despite this, maintains its essential role in the activation of *nif* genes during symbiosis indicating that this domain is dispensable, at least under this culture condition (Iismaa and Watson, 1989). Therefore, it is not so surprising that NifAp maintains a regulatory role during symbiosis, despite the lack of a N-terminal GAF domain. It is more surprising that NifAp is not functional during free-living growth. Our RT-qPCR analysis clearly showed that in free-living conditions, *nifAp* was expressed, which excludes the possibility that the lack of complementation of the Δ*nifAc* mutant is due to the absence of NifAp synthesis. It is possible that the GAF domain, which is dispensable during symbiosis, plays a more prominent role in NifA activity under *in vitro* conditions. We tested this hypothesis by constructing two chimeric NifA hybrid proteins containing the N-terminal part of NifAc and the C-terminal part of NifAp, but these constructs did not restore the free-living nitrogen fixing deficiency of the Δ*nifAc* mutant. Nevertheless, we cannot completely reject this hypothesis because it is possible that these hybrid NifA proteins did not have the correct conformation. Further studies at the protein level remain necessary to better understand the function and mode of action of both NifA homologs under free-living conditions.

The mechanism of regulations involving NifA in the DOA9 strain are certainly more complex than expected, considering that this strain also contains two *rpoN* homologous genes found on both the plasmid and chromosome. NifA activates *nif* gene expression and other genes by forming a complex with RpoN (Gong et al., 2007; Hauser et al., 2007). We can ask whether each NifA protein can form a complex with both RpoN proteins and depending on the NifA and RpoN composition, whether the activity of this complex and its affinity for a DNA binding motif differs. Intriguingly, while the promoter regions of *nifDKc* and *nifHc* contain a perfectly conserved NifA binding site (5′-TGT-N_10_-ACA-3′), only a nonconventional site differing by one nucleotide has been identified in the upstream regions of *nifDKp, nifHp* (Wongdee et al., 2016). Analysis of the expression of these *nif* genes suggests that these slight variations do not impact the ability of the two NifA proteins to activate the *nifDK* and *nifH* genes on both the chromosome and plasmid during symbiosis, since a similar level of expression of these different *nif* genes was observed for the 3 bacterial backgrounds tested (WT and the two *nifA* mutants). However, at the same time, under free-living growth, *nifDKc* was more highly expressed than *nifDKp*. Therefore, we cannot exclude the hypothesis that these slight variations in the upstream activating sequence differentially impact the affinity of NifA (at least for NifAc) according to the environmental conditions. Behind the control of *nif* genes, the NifA protein influences various cellular processes in rhizobia (Fischer et al., 1986; Gong et al., 2007; Hauser et al., 2007). Thus, it is attractive to speculate that the presence of multiple NifA and RpoN proteins in the DOA9 strain facilitates the switch from a free-living to a symbiotic lifestyle and vice-versa, allowing better control of the expression of various sets of genes.

## Author Contributions

JW, NT, NB, PT, and EG conceived the experiments. JW, NT, PT, and EG conducted the experiments. JW, NT, PT, and EG analyzed the results and wrote the paper. All authors reviewed the manuscript.

## Conflict of Interest Statement

The authors declare that the research was conducted in the absence of any commercial or financial relationships that could be construed as a potential conflict of interest.

## Supplementary Material

FIGURE S1 The two *nifA* genes in *Bradyrhizobium* sp. DOA9 strain are functionally redundant during symbiosis with *Aeschynomene americana*. **(A)** Comparison of plant growth (aerial part) non-inoculated (NI) or inoculated with WT and insertion mutant strains DOA9Ω*nifAc* and DOA9Ω*nifAp* (at 20 dpi). **(B–D)** Root nodules observed with a fluorescent stereomicroscope equipped with a green fluorescent protein (GFP) filter. **(B)** Nodules elicited by WT; **(C)** Nodules elicited by DOA9Ω*nifAc*; **(D)** Nodules elicited by DOA9Ω*nifAp.* **(E)** Acetylene-reducing activity (ARA) in *A. americana* plants inoculated with WT and insertion mutant strains DOA9Ω*nifAc* and DOA9Ω*nifAp*. **(F)** Number of nodules per plant elicited by WT and DOA9Ω*nifAc* and DOA9Ω*nifAp.* **(G-I)** Cross section of nodule elicited by WT **(G)** and mutants DOA9Ω*nifAc* **(H)** and DOA9Ω*nifAp* **(I)**. Scale bars are 250 μm for **(D–G)**. In **(E,F)**, error bars represent standard error (*n* = 10). Different letters above error bars indicate significant differences at *P* < 0.05 (Tukey’s HSD test).

FIGURE S2 *Bradyrhizobium* sp. strain DOA9 strain displays two distinct *nifA* genes. **(A)** Sequence alignment of NifAc and NifAp. Arrows and boxes indicate different portions of NifAp and NifAc used to form chimeric NifA proteins **(B)**. The color boxes indicate NifAc protein domains including GAF (blue), sigma factor 54 (δ^54^) interaction (purple), and HTH (green) domains. **(B)** Schematic representation of different versions of *nifA* introduced into plasmid pMG103-*npt2-cefo* under control of the constitutive *nptll* promoter. Each constructed plasmid was transferred into DOA9Δ*nifAc* cells for complementation experiments (see **Table 1**).

TABLE S1 Primers used in this study.

TABLE S2 Primers used in the qRT-PCR experiment.

TEXT S1 Analysis of Insertion sequences in the *nifAp* surrounding region.
