# Supplementary figures and images for: Regulation of Nitrogen Fixation in Bradyrhizobium sp. Strain DOA9 Involves Two Distinct NifA Regulatory Proteins That Are Functionally Redundant During Symbiosis but Not During Free-Living Growth
Source: Front Microbiol. 2018 Jul 24;9:1644. doi: 10.3389/fmicb.2018.01644 (PMC6066989; doi:10.3389/fmicb.2018.01644)

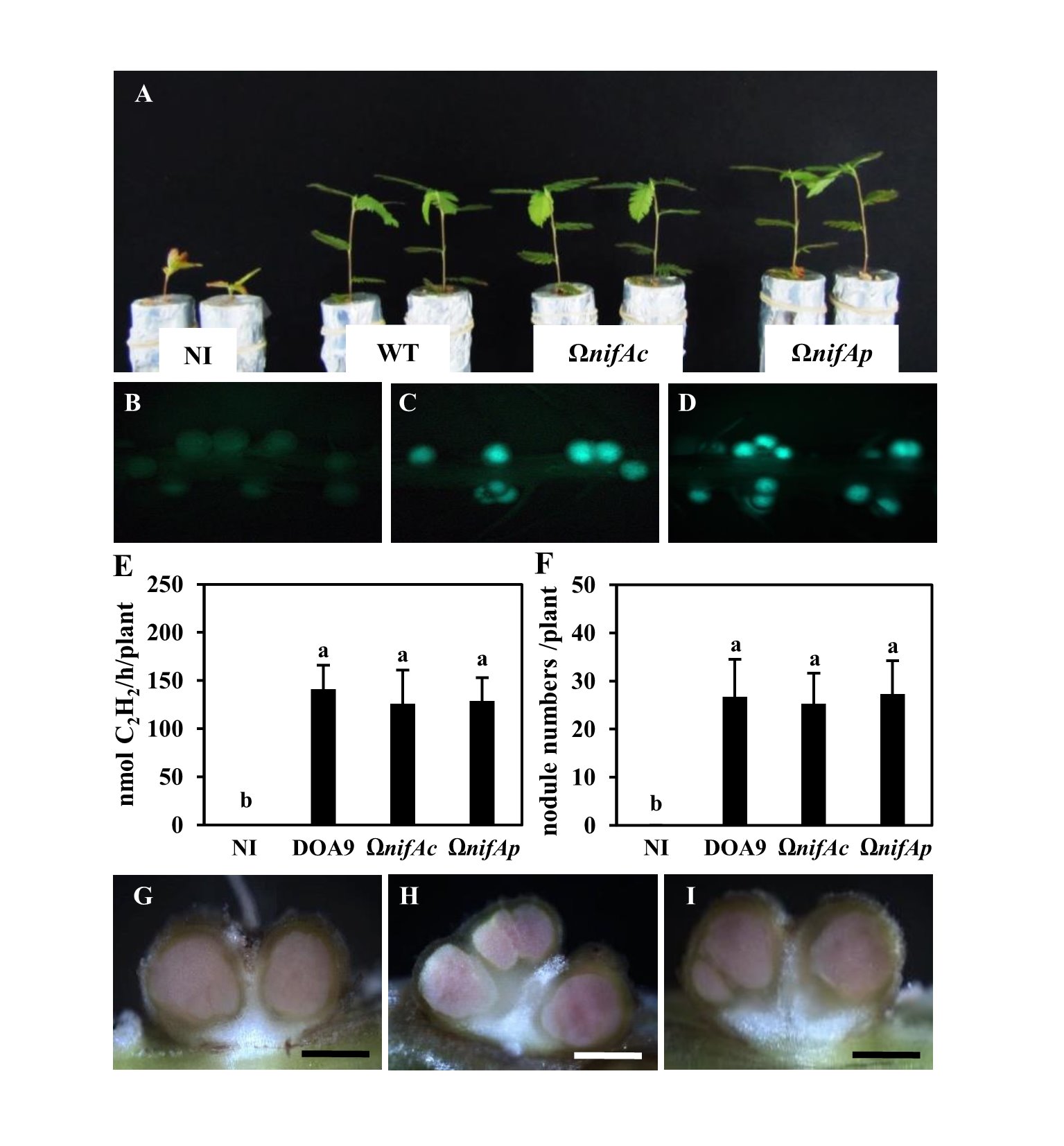

Supplement: FIGURE S1 — The two nifA genes in Bradyrhizobium sp. DOA9 strain are functionally redundant during symbiosis with Aeschynomene americana. (A) Comparison of plant growth (aerial part) non-inoculated (NI) or inoculated with WT and insertion mutant strains DOA9ΩnifAc and DOA9ΩnifAp (at 20 dpi). (B–D) Root nodules observed with a fluorescent stereomicroscope equipped with a green fluorescent protein (GFP) filter. (B) Nodules elicited by WT; (C) Nodules elicited by DOA9ΩnifAc; (D) Nodules elicited by DOA9ΩnifAp. (E) Acetylene-reducing activity (ARA) in A. americana plants inoculated with WT and insertion mutant strains DOA9ΩnifAc and DOA9ΩnifAp. (F) Number of nodules per plant elicited by WT and DOA9ΩnifAc and DOA9ΩnifAp. (G-I) Cross section of nodule elicited by WT (G) and mutants DOA9ΩnifAc (H) and DOA9ΩnifAp (I). Scale bars are 250 μm for (D–G). In (E,F), error bars represent standard error (n = 10). Different letters above error bars indicate significant differences at P < 0.05 (Tukey’s HSD test). [file Image_1.JPEG]

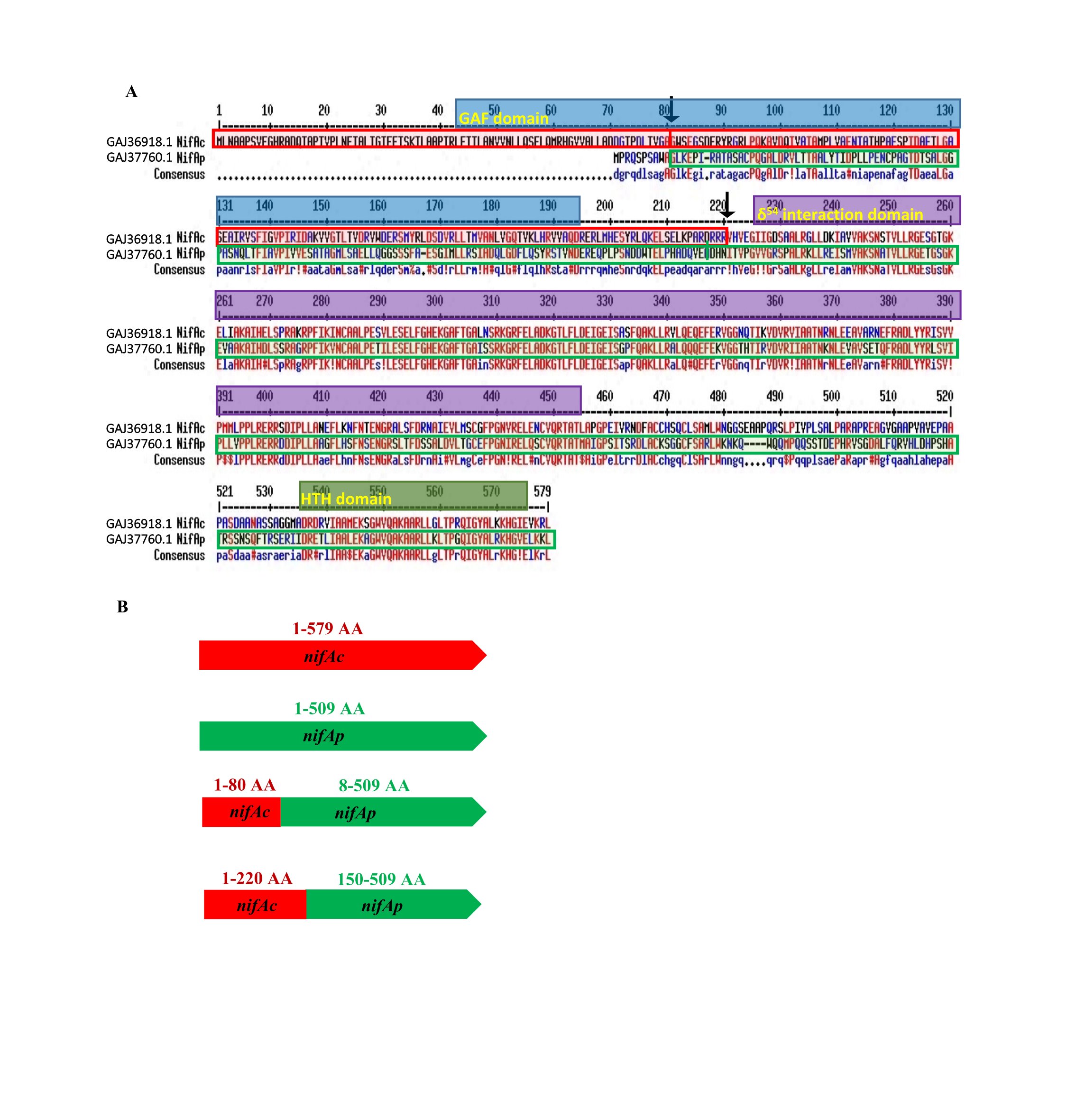

Supplement: FIGURE S2 — Bradyrhizobium sp. strain DOA9 strain displays two distinct nifA genes. (A) Sequence alignment of NifAc and NifAp. Arrows and boxes indicate different portions of NifAp and NifAc used to form chimeric NifA proteins (B). The color boxes indicate NifAc protein domains including GAF (blue), sigma factor 54 (δ54) interaction (purple), and HTH (green) domains. (B) Schematic representation of different versions of nifA introduced into plasmid pMG103-npt2-cefo under control of the constitutive nptll promoter. Each constructed plasmid was transferred into DOA9ΔnifAc cells for complementation experiments (see Table 1). [file Image_2.JPEG]
