# Supplementary material for: Regulation of Nitrogen Fixation in Bradyrhizobium sp. Strain DOA9 Involves Two Distinct NifA Regulatory Proteins That Are Functionally Redundant During Symbiosis but Not During Free-Living Growth
Source: Front Microbiol. 2018 Jul 24;9:1644. doi: 10.3389/fmicb.2018.01644 (PMC6066989; doi:10.3389/fmicb.2018.01644)
Supplement: TABLE S1 — Primers used in this study. [file Table_1.docx]

Supplementary Material

Regulation of nitrogen fixation in *Bradyrhizobium* sp. strain DOA9 involves two distinct NifA regulatory proteins that are functionally redundant during symbiosis but not during free-living growth

Jenjira Wongdee^1^, Nantakorn Boonkerd^1^, Neung Teaumroong^1^, Panlada Tittabutr^1a^*, Eric Giraud^2a^*

**Corresponding author**: [eric.giraud@ird.fr](mailto:eric.giraud@ird.fr) and [panlada@sut.ac.th](mailto:bbg.jane-na@hotmail.com)

# Supplementary Tables

**Table S1.** Primers used in this study.

36

| **Primers** | **Sequences (5’ 🡪 3’)** | **Relevant characteristics** |
| --- | --- | --- |
| NifA.DOA9c.in.f/  NifA.DOA9c.in.r | GTACCG**GTCGAC**GAGATCGCGCTGACCGGTATCTTC/ CAGTCG**TCTAGA**AGGCGGGATGGGTCGCAATATTTTC | Cloning of internal *nifAc* fragment in pVO155-Cefo-npt2-gfp after digestion of the PCR product by SalI/XbaI and the plasmid by SalI/XbaI. Plasmid constructed to obtain the mutant strain DOA9Ω*nifAc* |
| NifA.DOA9p.in.f/  NifA.DOA9p.in.r | AGCAA**GTCGAC**GACGTTCATCGCCGTTCCGATC/  GCAGC**TCTAGA**CTGCGACCGACAACGCCAGGTAC | Cloning of internal *nifAp* fragment in pVO155-Cefo-npt2-gfp after digestion of the PCR product by SalI/XbaI and the plasmid by SalI/XbaI. Plasmid constructed to obtain the mutant strain DOA9Ω*nifAp* |
| up.nifA.DOA9c.f/ up.nifA.DOA9c.r | GCGCC**GGATCC**GAGATCAAGGAGCGGTTAGACG/ ACAGTCGTCGAAGCTTGGAATCTCCGTTGCCCGTTAC | Amplification of the upstream region of *nifAc* and fusion with the downstream region by PCR overlap. The overlapping region is underlined. |
| dw.nifA.DOA9c.f/ dw.nifA.DOA9c.r | CGGAGATTCCAAGCTTCGACGACTGTCGCAGATGCGAC/ GCAAGT**CTAGA**CCGAGCTTCTCGTAGGTCTTGAG | Amplification of the downstream region of *nifAc*. After fusion with the upstream region, the PCR product was cloned into pK18mob-cefo-sacB using BamHI/XbaI restriction sites. Plasmid constructed to obtain the mutant strain DOA9Δ*nifAc* |
| up.nifA.DOA9p.f/ up.nifA.DOA9p.r | GGCGG**GGATCC**GTTTGCACCATGTCCGACGAATC/  CTCCGGTCAA**AAGCTT**CAAGACTTGGACACACCACGGAATG | Amplification of the upstream region of *nifAp* and fusion with the downstream region by PCR overlap. The overlapping region is underlined. |
| dw.nifA.DOA9p.f/ dw.nifA.DOA9p.r | CCAAGTCTTGAAGCTTTTGACCGGAGGCGAGCTGCAACTTC/  GGCGG**TCTAGA**GCAACCCTATTTCCTGTGTCTTGC | Amplification of the downstream region of *nifAc*. After fusion with the upstream region, the PCR product was cloned into pK18mob-cefo-sacB using BamHI/XbaI restriction sites. Plasmid constructed to obtain the mutant strain DOA9Δ*nifAp* |
| NifA.comp.DOA9c.f/  NifA.comp.DOA9c.r | TCGGC**ACTAGT**GATGTAACGGGCAACGGAGATTC/  GTTGCAGGAATTC**GGATCC**CAAGTACGATCATAGCTTCTTTAG | Cloning of the *nifAc* gene in pMG103-npt2-cefo using SpeI/BamHI restriction sites. Plasmid constructed to complement the mutant strain DOA9∆*nifAc* |
| NifA.comp.DOA9p.f/  NifA.comp.DOA9p.r | CATTC**ACTAGT**GTGTCCAAGTCTTGCCGAAAATG/  GTTGCAGGAATTC**GGATCC**CAAGTACGATCATAGCTTCTTTAG | Cloning of the *nifAp* gene in pMG103-npt2-cefo using SpeI/BamHI restriction sites. Plasmid constructed to complement the mutant strain DOA9∆*nifAc* |
| NifA.comp.DOA9c.f/ NifA1.hybrid.DOA9c.r (294bp)  NifA1.hybrid.DOA9p.f/ NifA.comp.DOA9p.r (1529bp) | TCGGC**ACTAGT**GATGTAACGGGCAACGGAGATTC/ TCAGTCCAGCAAGCTTGGCCCCGACCGTGAGATCCGGAATG  GGTCGGGGCCAAGCTTGCTGGACTGAAGGAACCGATCAGAG/ GTTGCAGGAATTC**GGATCC**CAAGTACGATCATAGCTTCTTTAG | The listed primers were used to generate the PCR products corresponding to a fragment of 294 bp of *nifAc* gene (1-80 AA) and a fragment of 1,529 bp of *nifAp* gene (8-509 AA). The two PCR products were then fused by PCR overlap and cloned into pMG103-npt2-cefo using SpeI/BamHI restriction sites. Plasmid constructed to complement the mutant strain DOA9∆*nifAc* |
| NifA.comp.DOA9c.f / NifA2.hybrid.DOA9c.r (714bp)  NifA2.hybrid.DOA9p.f/ NifA.comp.DOA9p.r (1112bp) | TCGGC**ACTAGT**GATGTAACGGGCAACGGAGATTC/ GTACCGTTATAAGCTTCCTGCGGCGGTCGCGTGCCGGCT  CCGCCGCAGGAAGCTTATAACGGTACCTGGCGTTGTCGG/  GTTGCAGGAATTC**GGATCC**CAAGTACGATCATAGCTTCTTTAG | The listed primers were used to generate the PCR products corresponding to a fragment of 714 bp of *nifAc* gene (1-220 AA) and a fragment of 1,112 bp of *nifAp* gene (150-509 AA). The two PCR products were then fused by PCR overlap and cloned into pMG103-npt2-cefo using SpeI/BamHI restriction sites. Plasmid constructed to complement the mutant strain DOA9∆*nifAc* |
