# Supplementary material for: Regulation of Nitrogen Fixation in Bradyrhizobium sp. Strain DOA9 Involves Two Distinct NifA Regulatory Proteins That Are Functionally Redundant During Symbiosis but Not During Free-Living Growth
Source: Front Microbiol. 2018 Jul 24;9:1644. doi: 10.3389/fmicb.2018.01644 (PMC6066989; doi:10.3389/fmicb.2018.01644)
Supplement: TABLE S2 — Primers used in the qRT-PCR experiment. [file Table_2.docx]

**Table S2.** Primers used in the qRT-PCR experiment.

| **Gene name** | **Gene description** | **Forward/ Reverse (5’-3’)** |
| --- | --- | --- |
| *nifAc* | Nitrogen fixing regulator gene located on DOA9 chromosome | GCACCCCGGCGACACCGGCTTTG/ GGTGTCGAACACGTCGCTATTG |
| *nifAp* | Nitrogen fixing regulator gene located on DOA9 plasmid | CGGTGCTCTTACGAGGCGAGAC/ CCAGTAGCGGAATGACGCTCAGG |
| *nifDKc* | Operon of nitrogenase structural genes on DOA9 chromosome | GGGTCGGATGCATCAAGCAAG/ GTTGCTAGGCTCATACGAATATC |
| *nifDKp* | Operon of nitrogenase structural genes on DOA9 plasmid | CTCGCAGCGCCCTCGACAATAG/ CTTCTCCGGATAGACCTTCAAG |
| *nifHc* | Nitrogenase structural gene on DOA9 chromosome | CGACCACGTCACAGAACAC/ CCTTGTAGCCGACCTTCATG |
| *nifHp* | Nitrogenase structural gene on DOA9 plasmid | AAGGGTGGTATCGGCAAATC/ GCAGCGGATGTTCTGGTAG |
| *dnaK* | Housekeeping gene use as internal gene in this experiment | GAAGTGCTGCGCATCATCAA/ TCCTTCTGGAATTCGTCGGC |
