# Supplementary material for: Regulation of Nitrogen Fixation in Bradyrhizobium sp. Strain DOA9 Involves Two Distinct NifA Regulatory Proteins That Are Functionally Redundant During Symbiosis but Not During Free-Living Growth
Source: Front Microbiol. 2018 Jul 24;9:1644. doi: 10.3389/fmicb.2018.01644 (PMC6066989; doi:10.3389/fmicb.2018.01644)
Supplement: TEXT S1 — Analysis of Insertion sequences in the nifAp surrounding region. [file Data_Sheet_1.docx]

Supplementary Material

Regulation of nitrogen fixation in *Bradyrhizobium* sp. strain DOA9 involves two distinct NifA regulatory proteins that are functionally redundant during symbiosis but not during free-living growth

Jenjira Wongdee^1^, Nantakorn Boonkerd^1^, Neung Teaumroong^1^, Panlada Tittabutr^1a^*, Eric Giraud^2a^*

**Corresponding author**: [eric.giraud@ird.fr](mailto:eric.giraud@ird.fr) and [panlada@sut.ac.th](mailto:bbg.jane-na@hotmail.com)

**Supplementary Text S1**

**Analysis of insertion sequence**

**From >PlasmDOA9_398416 to 402975_+1 at upstream of nifAp**

BlastN in <https://isfinder.biotoul.fr> to find IS type


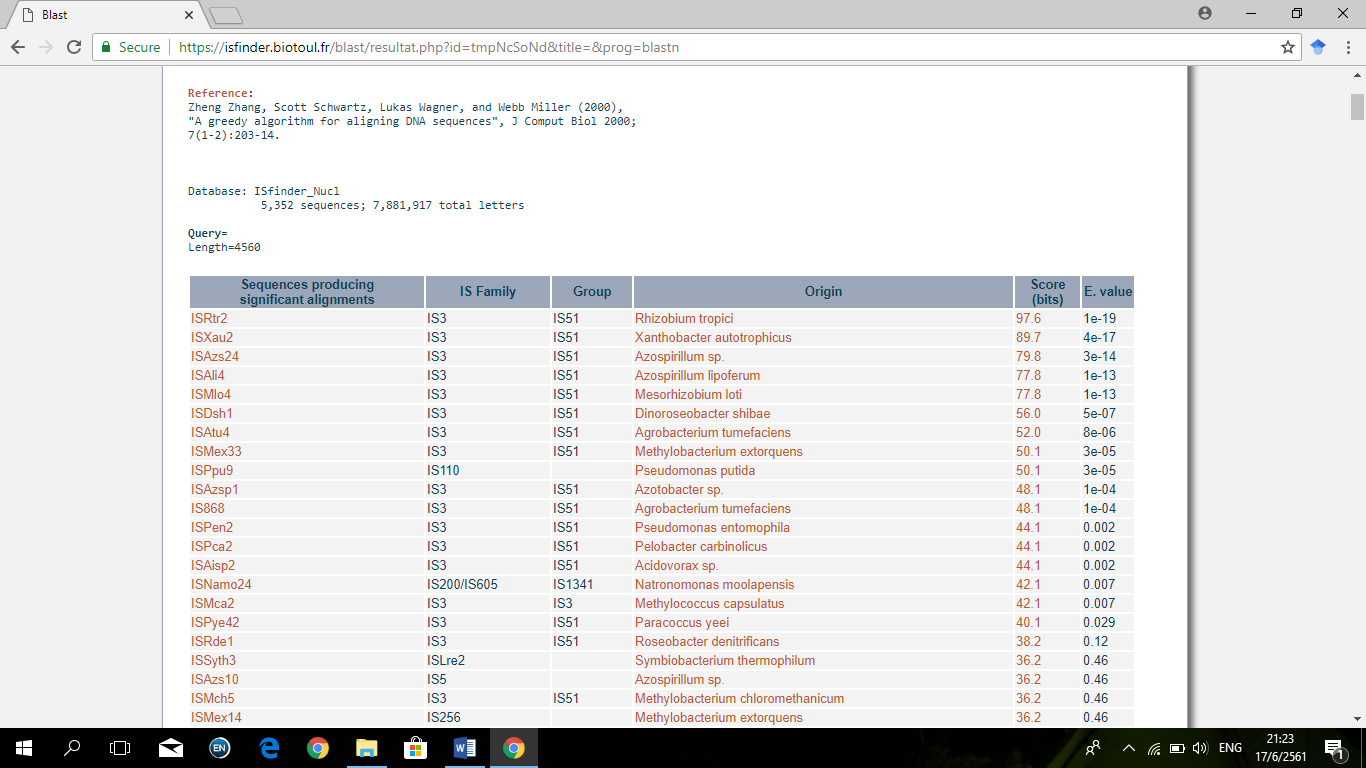


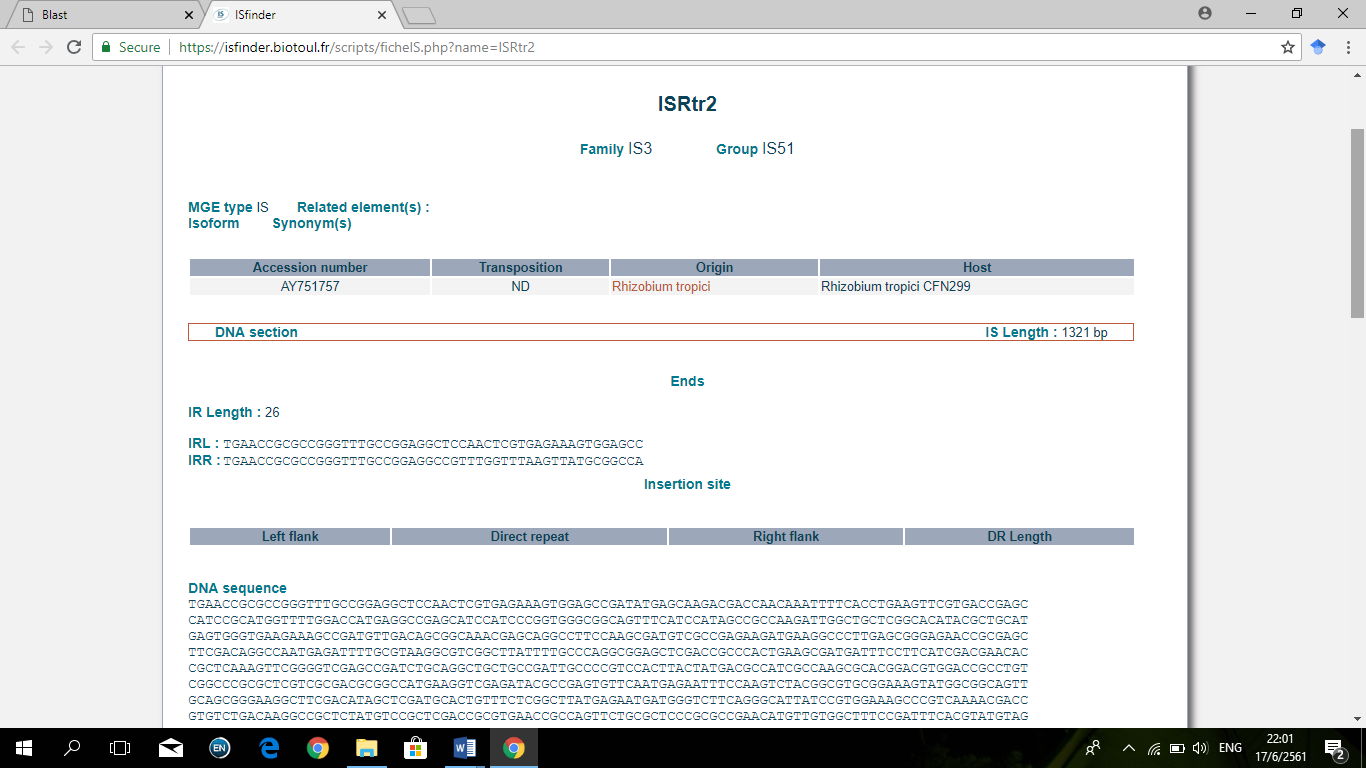


TGAACCGCGCCGGGTTTGCCGGAGGCTCCAACTCGTGAGAAAGTGGAGCC --> **IRL**

TGGCCGCATAACTTAAACCAAACGGCCTCCGGCAAACCCGGCGCGGTTCA --> **IRR (reverted)**

>PlasmDOA9_398416_402975_+1

ATGAAACAATCTCTTGCAATTTTATCATGTTTGGTTTGCGGTTTGCTCGTTTTGAATGGG

ATCGCACTCGCAGACTCAATGCGACGCTACAGCTGCACCGTAGAGGGGTCCGATCGAGAG

CCGGTCGGTGATCGGGACGGGCACCTTATTGTCAGCCTCCAGTACACCTGTCACGTCGCC

AATGGAGCGCTGAAGGACTCGGGAATTACGGGACTGTTCGTCAGCGAATGGAGCAGCGAG

AAACAAACGTACTTGGCTTCTCTTGACGTTCATCGCGCGCTCGACGGATTTGCGGTTAGT

CAGCTCCTGGAGGGGATCGGCTCCTCCCTTATGGAGGACAACAGGGCTGCCGGCATTGCT

GCTTCCGGCAAGACTGTGTTTAAGTTTGCCTCAGGCTCGTTAGCGGTTCTCTCAGGAAGG

ACTGTGACTTTCACGACTAAGCCCCTCGACTATCGTCAATTCGAGATGGAGTTCACGGAT

TGGCCTGACACTATTCAGCCTAAATGAACCTGCTGCCGTCGCACCAAGGAT

TGAACCGCGCCGGGTTTGCCGGAGGCTCCAACTCGTGAGAAAGTGGAGCC --> **IrL**

TGAACCATGCCGGGGCTGTCGGAGGCCAGTTTGGTTTAAGTTATGCGGCC --> COMPLEMENTARY

ATGCCTTGGGGGCCGGCAT

GGCGTAGTAGCGTTGCTCAGCTTCGACCGGCGGTATGTTGCCGATGGGCTCGAGGAGCCG

CCGGTTGTTGAACCAATCGACCCATTCTAGGGTGGCGAACTCGACAACCTGGAAGCTGCG

ACATAAGCGAAGTCGGAGAGCCAGGGGACATTCGGTCGTGGCTCCTTGAACTGGCGGTTG

ACGTGATCAGCGGGCAGGGCGCGGCCTTGTCGCTGAGCGTGGGCGGGCTTGCCGCGGATC

CCCCTTGCAAATCCATGTCCCGCATCAGCCGCGAGCCCGTGCAACGGGCAACATCGGCGC

CTTCGCGCTTGAGCTGTCGCCGGCAGCGTCGTCCTATTCAGAAAACAGCCGATGACATTG

AGCGGAGTTATTGGGCGCGCTTCTTGGGAAGCGAGCCGCCATTCAGCGCATCACCACACG

CTGCCGTCACGCCATGGGCCAGCCCAGTGCACCCTGCGAGAGCGATTCGATCCCGCCGCC

ACATAAAGCCAGCCCTCGACTGCCCAGAGATAGGCGAAGTCGGCGATTAGTTGTAGTACG

GCCGCTCAGCGGTAAACTCCCGATCCAGCAGATGCGCCGACACGATCTCGGCTGCCGACC

CCCGCCATTCATAAAGCGCCGGCGGGCCCCGCGCCCGCAAGCCCTGCAAGCGCATCAGCC

GCTGCTCGACCCGGTGCAGGGCACAATTCGCCCCGTCAGCCAGAAACTCGCGCCAGAGCC

GGCGCGCGCTATGAGTTCAGTCGCTGACGGCGAAGCTGGCCTTCACTTTGCCGCCGAGAC

CAACGTCCTGCGGGCCCTGACGCTTGGTGAGTGGTTACGCCCATTGAAGCTCGACCGCGA

TACCCCCAGCGCATCGCATAGCCATGCCACCGGCCAGATTGTCCGGCGCCTCGCGATGAA

GACGAACTTCATGTCGCTCCTTCGCGAAGACGGCTGCGGCCTTCCTAAGAAACAGCCAAT

GCCCGGGTCCACTTGCAGGACGAAGCCGAGTGACTTGGCACGTCGCCTCAGATTGGCGAC

GACACGGCCGCGATATTGTTCTTCGTAGTGATCAGCACCCGGATCCCTGTAGGTCATGCC

GTGCCGGAGCGTATTGTAGAACAGAACTGCTATTTTGCGAGCGGTTGCCGTCACCGCGTT

TGGCTTGCCGGCGCGCAAAGCCAGCCGTCGATAAAATGCGCCAAGCGCCGTATCGCTCCA

CCCAACCGAGGTCGCTGCAAGCCGCAGCAGTGCCGCTGCCCGACTGGACGATCGCCGCAT

GCGCGAAGATAGTAGCTTGCCACCGGAGATATTGTTTGGGCCTCCCCTAAATCGGTGGAC

ACCTATAGCTCAGGCAGCGAGAGGTGTCAGATGGCGAAACGGCAGCGTCGGTCCTATTCG

GACGAGTACAAGCGACAGGCAGTTGATCTGGTTGTATCGAGCGGCCGCTCGGCGAAGTCG

ATATCGAAGGAGCTCGGGCTCGACGGCTCCGTGCTCAGCCGATGGGCGAAGGAGCTGGTC

ACAGTGGCAGCGGGCGGCAGCGCCGCGGCGCCCACGCCGCAAGCGGCGGTGCCGTCGGCG

GACCAGGCTGACATGATCGCCAAGTTGCAGCGGGAAAACGAACAGCTGCGCATGGAGCGC

GACATTTTGAAAAGGTCGATCGCGATCTTTGCTGGACCCCGGACGAAGGGTTCCGCTTTA

TCGAAGATCGCCGTGCGGACTAATCCGGTTGAGGTCATGTGCCGCGCGCTCGGCGTCTCG

CCTGCAGGCTATTAGGCGGTCGCGCCCGGAAAGCCTCCGGGCCTTGGCCAATCGAGAGCT

GCTGGATGCTATTAAGCGGGTTCGTCGCGATAGCCGTGGTCGCTATGGCAGCCCGCCATC

CAGCCGAGGACGCCGTATAGCGCTGTCCTGACGTCGAACGACGGTGTGTTAACCTGCTTG

GTCTTTACCCGCGGCCTGGAGAGCTCGCCGATCGGCTTGGCTCCTTTGTCAGTCAGCGCC

GTGATCAAGGATTCCAGCTTGCGGTCACAATCGAGCATCTTGGCCTGGTAGGTGTCGTAG

AGCTCCGGCGACTGGGTCAGTGCGAAGACAAGTTCGTCCCGATCGTTGCCTACCAGTGCG

GCGCGGATGGTCTCGATGGTGTATTGACAGCGCACGTCACGGTAGGTCGCCAACACGTCA

GCATTCCGCTCGCCTGCCACAATAGCTCGGATAATCCGCATCCCGGTCGCGCCCGTGGTG

TCGGAGACGACGTGATGAAGTTGCAAGTTCATCTCCATCAGGGCCTTCTGCATGTGCTGG

ATATGAGCGGCTGCATATTCGACTAGCCGCTCTCGCTGGCGGAGATAGGCGCGTAGCGTC

GCAATATCGGCATCAGGCCGGAAGCTGCCGCGCAGCAAAACCACAGGAATGAAGCTGGCG

CAGCCAAGCGACGTCACTGACATCAGTTTTTGCGTCCAGGCACGCTCTTGGCGTAACGTG

CGTTGACCAGAATGACGTCGAACCACGCTGCTCGAGAATCTCGTACACCGGGATCCAATA

GACCCCCGTGGATTCCATCGCGACACCGATC

ACGCCGCACGCTTTGAACCAATCCGCCACTTCGTGCAGGTCCTGCGTGAATGT --> COMPLEMENTARY

TGGCCGCATAACTTAAACCAAACGGCC---TCCGGCAAACCCGGCGCGGTTCA --> **IRR**

GCCAAATGCACGCACCGGCGTGCCGGTGCCGCCTGG

GTCGACCGCCGCCATGTGCATTTTTGACCCTATGTCGATGGCGGCGGCTTCAACGTTGAC

CGGCTTTAATCCGGGCGGCCACCGGCAGGCGTCTTCGGCATTGCAACTCCTCCATCCAGT

CATGGGGCTGGAGGGTCTGGGTTATGCCAATCGTCATCCTCCAAATCGGGATCGCCGTCC

GAACAGCGTCACCATTCTCACGTTCCCATCCACCCATTGGCCTCCAGCTTGGCGACCTCA

CGGCGCTGCGGCTCGATCTCTTGCTGCTCGGGCTTCATCTGCCCATGCCGGGGAACGCCT

GTACCGGGTCAGGGCCGAACTCCTTCACCCATTTGCGCAGTACGTACTCATGAACCCCCC

GGTCGCGGCCGGCTTGCGCCACCGACACCCCGCGCTCCCTGACACGCTTGACCGCTTCGA

TCTTGAACTCTCGGCTAAACTTCCGTCTCTGCATGGCCAACCTCCGGCTTGATAAAACAC

CCAATCTCGGGTCCATCAAAACGGCAGCAGCTCACTGCCTGCTATCAATTTTGGCGGAAA

CTAGTTTGCACCATGTCCGACGAATCTAACCAGAGTCGGGATCTGTAGTAAGGCTGTTGC

AAGCCCTTTTCCGTTACTAATTGTTACATGCCTTGGCAAAGCGGTCTTTCGCAGGGGTCG

TGGAGCGTACGCTCCCGGCGTCCAACTCATTGCGCCGAGGCGGTTACATGATGCCCATAG

GAGGCAGAGGCATCGATGGCCGAGCGCTTGCAACCCCTGAGGGCAAGTCGCGGTTCGCCT

CACACATGCGCCAATGTGGCGCACCCCTTGGATTGAGTGGCCTCACGCAGCCAAATAGGC

GCGAGAGGCTCGCCCGGCACGTGGCAATTGGGATGAGTAATGCCCGATTTTCGCGCCTCA

TCGCACCTGGAGAGAACCCGATGGGGGGGAGCTGCACCGGGGCATATCCTCCCGTGGGGA

CCGGATTGTTAATGCTAGTTGGCCTTGTCGCTGCTCTAGGAACGTTGTTGTTAGGTCCGC

GGCATCGCAGATACCAAACGCCGACGTGACACCACTGGTCTCGAACTACGAATTGGCCAA

AAACACGACATATGACCGGCAACCCTGCTTTTCTTTCGGCGGCCAACTCCGACTTATCCG

GAGCGGGGCCCTATCATCTTAGCCGTCCGCCAAGCCTGGACGCGCCAGAGATCTTGCTGT

CGATCGCAGAACAACTCGCCGCACTCTATCGGCTCGAGCTCGCGCTTGAGGCGGTCGCCG

GGTTAATACAGTCGCGGCTTGGGATGCAACATTCCGTGGTGTGTCCAAGTCTTGCCGAAA

**From >>PlasmDOA9_404488 to 408844_+1 at downstream of nifAp**

BlastN in <https://isfinder.biotoul.fr> to find IS type


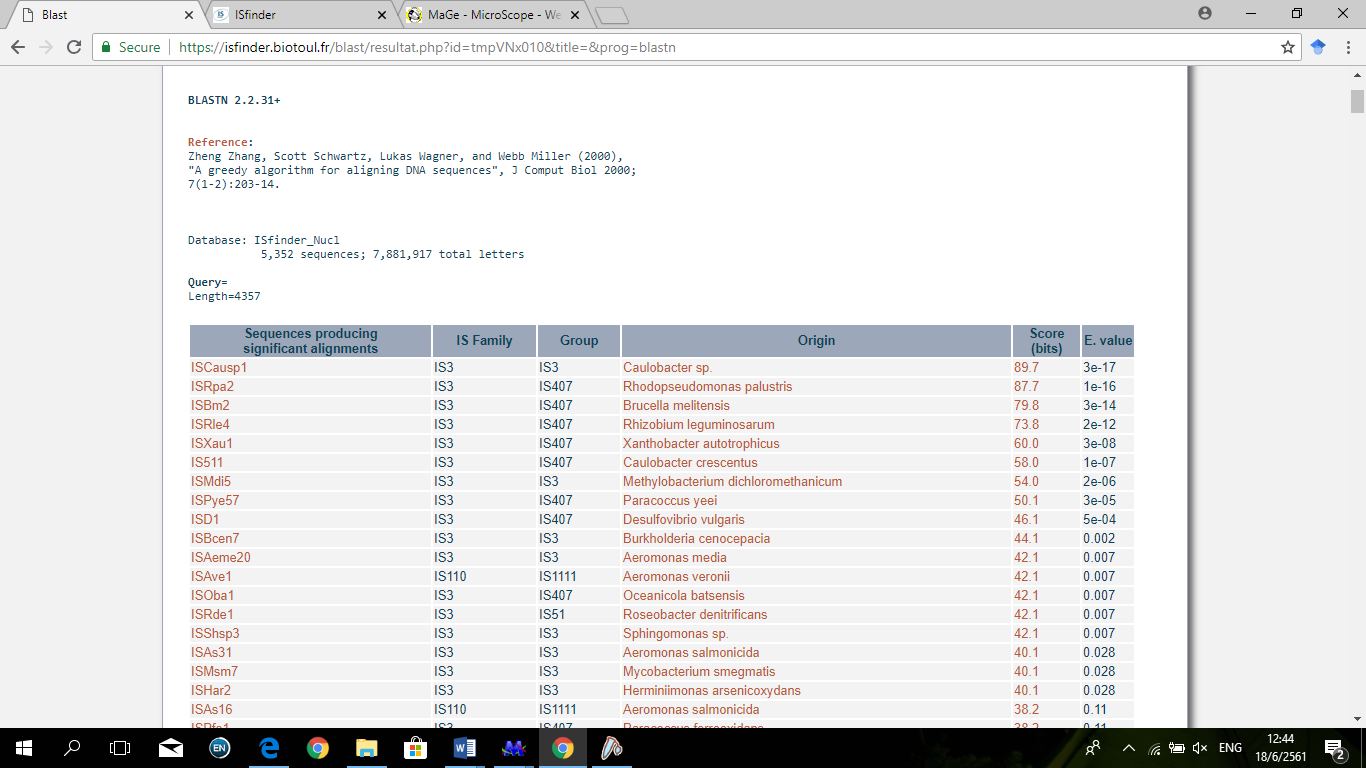


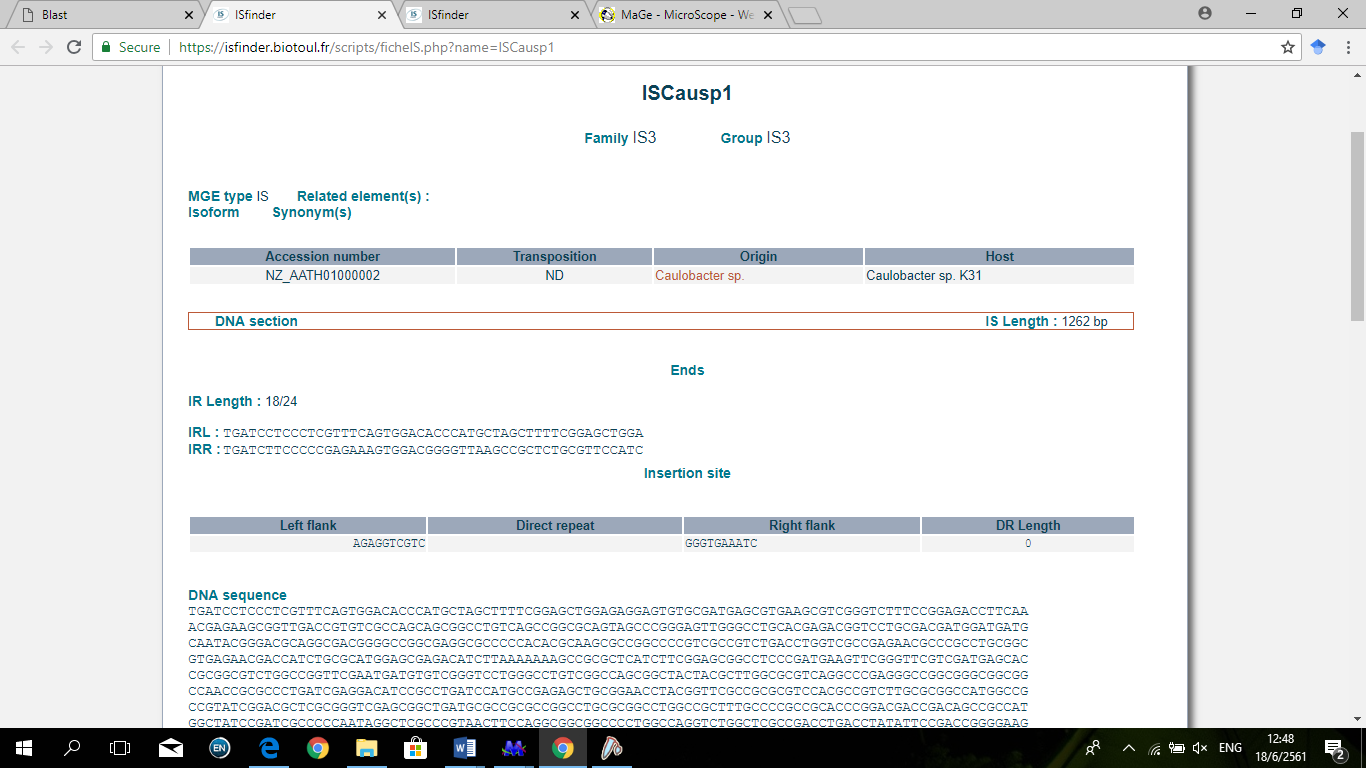


TGATCCTCCCTCGTTTCAGTGGACACCCATGCTAGCTTTTCGGAGCTGGA --> IRL

GATGGAACGCAGAGCGGCTTAACCCC-GTCCACTTTCTCGGGGGAAGATCA --> IRR (reverted)

**From >>PlasmDOA9_404488 to 408844_+1**

TCGTACTTGACCGGAGGCGAGCTGCAACTTCATCAGGTCTGGTTTGTAGGCATGAGATCT

CACCTTGCCCCGGCCATAATCCAGTTCGAAGCTCGTTTCGGCGCCCAGTGAGGACCGGAA

GGAGCATGAGGCTGACGCTGGTCCGTGGCGGAAGCCGAGCGCAAGCGACGCCAGCATCTA

CCAGTGGAAAGCCAAGTTCGGCGGAACGGACGTCTCGGAAGCCAAGCGGCCGAAGACGCT

GGAGGACGAGAACACGCGGGTCAGGCAGGCGAATGATCATGCGCTTCGTGCCGATTAAGA

CGCTGGGATTGACGCCACAGTCTCATTCAAACGGAGGCAAACAGCGGCTGGGACGAATAC

CGAAGATGGCAAGTCCGGAATTCCGCCCTCTCCTCGTTGCCAGGGAACGGCCGTCTTGCG

GGTTCTCCGAACCGGCGCTTGGGCGCGCCGTGGCTGAAGGCGCTTCTTGCCAGACGGCCC

TTTAAGGTCGTTGCTGTCCCGCAGGCCAATAAGATGAGCGGATCACCTGGGCGCTCCTGA

ACGAGGGCGAAATTGGCGTCCTGACCCCCAATAGCGATTACCGCGGTCGCCGTCTGATGG

ACGCCATGACCGCCAAAACGATCTGATCGGCGCAGGCCGGCAGAGGCAAGGTGCAACAAC

AGGCGATGAACCCATGGGACGAAATCCCGAAAGAAACGTGGCACTCACCGAATGTCGGCC

TCGACGACACTCTGGAGTACTGTCGCCGCCATAAAGGGCTCTTATTGTGCTACCAGGTAG

ATGCGGCAAGACACAGGAAATAGGGTTGCAACGGCCCGCCATGCCGGTTCGGCCGACCCA

TTGAGAATGTGGATGCCCTTTATCATAACAATCGATTTCACCGACGCCGGTCAAGCTTGC

TACTGGTGACACGGCGTCGGATCTGCTGAGAGTTATACGTTGAGAAATCGCTCCCGTGAC

GGCGTCGCTTCGGGAAGGCTCGCTGGCCTCCAAACGGAACCTTATAACGAGTGCTCATGA

CGACCATGATGAATAGTATCAGAAACGCAACCTCGACGGAAATAATGGGAATGCGAGATC

AGGCATTGGCGTGGTGGCTACCGAAGCTGAAACACTATGCATCTTGTTTGCGGCTGCTAG

AGAGCGCCTTACCCGTTCGTCGGCCTCACCTTGAGTCGCTTGCTGACCCGCCTGCTCCGG

TCGGTTGCAGCACTTCTCATGAACATCTCAGGCAAAGTATCGAGTCGCGAATCGCAGAGA

TTGGAAGGTTAGTAGGCGAAGGTGAAAAGCCGGTCGAAGCAACCCGGCATCTCGCTGACC

TGGCGAACGCGCTCATATTCGCGCAGCTCGGCATCTTTTCCAAAGTCAAATTGGTCAGTA

CCGAGACAGCCGGCGGCCTCACGAACCGTGCTCCCTTAGAGACGAGCGGGATCTATGAAC

CGGATGCGCTTTTGAGGCTTGGCCATTTGTTCGACAAAGCTGTATTGGCGTTAACTCCGA

ATTTGCGAACGCAAGCCAATAAAGCAGAGATCGCAAAACTTATCCTCGGCCGGGCATCTG

AAAGCAAAGCCGAGCAATGTCTTTTCATTAGGCTTGTCATTGCGATTGTTGCCGCCGCCG

AAGGGATGTAACGCCTGGCGGCCATTGTACGTTACGCAATATGATTTCGGCACCCGCAAA

GCAGCTGTCGAGGTGCTCAACCTCGGCATTTGCTGTGTCTCCCGCCGTGGCGAGGTGCGG

CGAAGCCAGACC

TGATCCTCCCTCGTTTCAGTGGACACCCATGCTAGCTTTTCGGAGCTGGA --> IRL

TGAGCCTCCCCTAATTCGGTGGACACCGAGCTTAGGCAGCAAGAGGTGTC --> COMPLEMENTARY

AGATGGCGAAACGGCAGCGACGGTCCTATTCGGACGAGTACAAGCGGCAAGCAGTTGA

CCTGGTTTTGTCGAGCCGGCGCTCGGCGAAGTCGGTCTCGAAGGAGCTCGGGCTCGACGG

CTCGGTGCTAAGCCGATGGGTGGCGGAGCGGGGCACCGTGGCAGCCGGCGGCAGCGCCGA

GGCTCCCACACGGCAAGCGGCGGTGCCGTCGGCGGACCAGGCTGACGTGATCGCGCGGCT

GCAGCGGGAAAATGAGCAGCTGCGGATGGAGCGCGACATTTTAAGAAAGTCGATCGCGAT

CTTCGCTGGACCCAAGACATGAGATTCCGCTTCATCGAAGATCGCCGCGCGGATTATCCT

GTCAAGGTCATGTGCCACGTGCTCGGGATCTCTCCTGCAGGCTACTATGCTTGGCGGGCG

CGCCCGGAAAGCTCTCAGGCCGCTGCCAATCGCGAGCTGCTGGAGAACATCAGGCGGGTT

CATCGCGACACCCATGGTCGCTATCGCAATCCGCGCATTCATGCCGAACTCAAGGCCCAG

GAACACCATGCAAGCCGTGGCCGGATCGAGCGGCAAATGCGCTGCCATCTCGGCCGGCCT

GCGGCGATGCCGCACCACCGATAGCGGGCATGGCTTCCCGATCGCGCCAAACCTGCTCGA

CCGGAACTTCTCGGCCGCCATGCGCAATCAGGTCTGGCTCGCCGACATCACCTATGTCTG

GACCGGAGAGGGCTGGCGTACCTGGCCGCCATCATGGATCTGTGCACTCGCCGTATCGTG

GGTTGGGCGATGGACGAGCATCTGCGCACCGAATTGCCGCTCGCCGCATTACGAATGGCC

ATCAAGGGACAAGGGCCCGGAGCCGGGCTGATCCATCACTCCGATCGCGGCACCCAATAT

GCTTCGACCGAGTACCGCCAGGCGCTGCAAGCCGCCGGCTTCCGGCCCTCGATGAGCCGT

AGAGCCGACTGCTACGACAATGCCCCGATGGAAAGTTTCTTCCATACCCTCAAGACCGAA

CTGGTCCATCACCGCAAATATGCAGCTCGCGATGAAGCACAACGCGACATCTTTGCCTAT

ATCGAGGGCTTCTATAATCAAACCCGCAGACACTCGGCCATCGGCTATATCAGCCCGATC

G

AGATGGAGCTAAAATCGGCTTGATCCCAGTCCACTTTTTTGGGGCAAGATCA --> COMPLEMENTARY

GATGGAACGCAGAGCGGCTTAACCCC-GTCCACTTTCTCGGGGGAAGATCA --> IRR

GGCCGCG

CTGCCACCGCGTCGCAGAGGGCACCAACCTCAATTCACTCCGCTCCGCTGCTAATGGATA

GCACTTCGGCGCGAGGTCACTGAAACACCGCTTCATTTTCCACATGCCATCCGAGGTACG

TCTTGCGGCGTTGTGCGATCCGCTCATGGCCCTGGAACCGGAAAAGCTCGCGTGTCGAAT

CAGGAGAGAAGCCAGAGGATAGCACGAAAGATAAGTATCCACGTTGTAAGGCAGAAAAGT

ACCCCGAACAGCCATACTCTCGCTCTGAATCGGCGGCGCCAGTTTACAAGTTCCGATGCA

CAACCGTTGAAGCGTCGACCGCGGACGACGTCAATCATTTCCCGGTAATTGCCAAAAACA

AAATATGAGCGATTACTTCGGATGTACCTTATCCGATCTTGAGGTGAAAGGCACCATTCC

CAGACGTACTGCGCCTCCTCCAGCACACTGTCCAACCTTGGCCTATACTTTCGGGAGTTG

GGTGCACGCCGCTCAATGAGCGGCGAGTTGGAGTGAAGCGATCGCGGGCTCTCGGTAAGC

GTGAACATTTGCCGAAACATCAAAGTGAACCTTTTCGAAATACTTCGGACGATGCGTACA

ATGTGACGTAACGAGAGAATCAAGAGGATCAGGCGCAGTGCATCATCTATTTCTGTACAA

GGCCGGTGGCGGGGACCTCGAGTTCGAGTGCCAGCACCGGATAACGGCCGGATGTGAGCA

CGGGTACTGTGGCCGGCATAGGCCGGGCGCCGCTGGCTAACGGACTTTCAGCTCCTGAGG

CAGCAGATAGTGCGTATATTGTTTATCGTATAAGTAGCGGCCAGTTGAGCTAGCCCCCGG

GAACGTAGGACATGGGCGATGCGGCCTCGCCGAGAAAGAATGATCAACTGATTAGGAAAA

GGCTTGAATCGCACGCGACTGGAGATCGTACGATGCAGGGAAATGGTGAACTGCGAGACG

AGTCACAGGAAGCGGTGACCCAGCCCGGATGCCCCTCAAACTGCCGCTCGTGGCGCGGCT

GACCGTGTAGGCGGCTGCAAATGGACAAGGTTTGAATGGCTCAAGATGAACCGCTTGAAC

GTTATTGTCGTAGCACTTGTATACGTTCAACTGCACCTGCACACGATATGACGATACCGC

GCCAGCACTCAACGGTCCTAACCTCCTAGAGGACATAATGCATCAAAGGCAACTCAACTG

GTGCCTCTGGATCTGTAAAATGAACTGCTGTTTGATCGCGTTCAGTATTGTTGCAGCGAT

GAGCATTTTGTCAGTACGGTGTGCCAAAGCCAACGAATCGCTTCCAGCTGCGCAGGAAAC

GCAGGCGTGTATGCCCGAGCGTCGTTCGTTTCTGTAG
